# Quantifying short-range order using atom probe tomography

**DOI:** 10.1038/s41563-024-01912-1

**Published:** 2024-07-02

**Authors:** Mengwei He, William J. Davids, Andrew J. Breen, Simon P. Ringer

**Affiliations:** Australian Centre for Microscopy and Microanalysis, and School of Aerospace, Mechanical and Mechatronic Engineering, The University of Sydney, Sydney, New South Wales Australia

**Keywords:** Metals and alloys, Characterization and analytical techniques

## Abstract

Medium- and high-entropy alloys are an emerging class of materials that can exhibit outstanding combinations of strength and ductility for engineering applications. Computational simulations have suggested the presence of short-range order (SRO) in these alloys, and recent experimental evidence is also beginning to emerge. Unfortunately, the difficulty in quantifying the SRO under different heat treatment conditions has generated much debate on the atomic preferencing and implications of SRO on mechanical properties. Here we develop an approach to measure SRO using atom probe tomography. This method balances the limitations of atom probe tomography with the threshold values of SRO to map the regimes where the required atomistic neighbourhood information is preserved and where it is not. We demonstrate the method with a case study of the CoCrNi alloy and use this to monitor SRO changes induced by heat treatments. These species-specific SRO measurements enable the generation of computational simulations of atomic neighbourhood models that are equivalent to the experiment and can contribute to the further understanding and design of medium- and high-entropy alloys and other materials systems where SRO may occur.

## Abstract (web summary)

A method is introduced to quantify short-range order in multicomponent alloys using atom probe tomography, which enables further understanding and materials design related to atomic-scale solute engineering.

## Main

Short-range order (SRO) is used in crystallography as a quantitative measure of the relative tendency for the constituent elements in a material to deviate from a random distribution. Specifically, it is a measure of the tendency for certain species to exhibit particular short-range nearest-neighbour (NN) relationships^1,2^. These relationships may be random, preferred (‘clustering’) or non-preferred (‘anti-clustering’). Discussion, computational simulation and experimental analysis of SRO has been prominent in research on conventional dilute multicomponent alloys and, more recently, in the so-called medium- and high-entropy alloys (hereafter referred to as M/HEAs)^3,4^. The latter have attracted enormous research attention in recent years due to their potential as a gateway to new alloy classes with outstanding properties in various realms including mechanical, thermal and neutron irradiation shielding^5,6^. In conventional alloys, there exists an extensive literature demonstrating that SRO or solute–atom clustering can enhance strength and that this generally occurs with little or no concomitant loss in ductility^7–9^. In M/HEAs, the subject is controversial, but there is also an emerging conjecture that SRO influences the microstructure and mechanical properties^10–13^. Research on M/HEAs has kindled two intertwined controversies. On one hand, there is the question as to whether we can reliably and accurately measure SRO in multicomponent alloys^10^. On the other hand, there are questions around the degree to which SRO might influence the mechanical properties of M/HEAs. Embedded in these controversial questions is the assumption that SRO actually exists, that non-random NN arrangements occur and that this SRO can be engineered via materials processing. Our contribution is focused on the first of the above two controversies and tests the embedded assumptions.

Examples of this controversy or inconsistency related to SRO measurements in M/HEAs abound, and the situation remains unsatisfactory^11,14^. In essence, the emerging conjecture from certain recent works^11^ is that changes to the SRO not only affect the planar slip and yield strength, but that they enhance the work-hardening capacity during plastic deformation. Recently, however, the alternate conclusion has also been posited^14,15^. A resolution to this question is important because of the potential to leverage SRO as a state variable of the microstructure that can enhance the mechanical properties in M/HEAs, as is already applied in certain conventional alloys. This would be a dramatic breakthrough in the design and development of this emerging class of materials.

A rich body of literature surrounds the quantification of SRO in materials systems. Most work has been scattering based, primarily using X-rays, though neutrons and electrons are also widely used^11,12,16–20^. In brief, we summarize that scattering-based SRO measurements are plausible for dilute binary systems, though major experimental challenges remain for multicomponent systems because of convolution issues in the scattered signal from different atomic species. In particular, the difficulty of separating background effects from the SRO signal in the diffracted intensity is a challenge. Diffuse scattered intensity and superlattice reflections are variously offered as evidence of periodic SRO (that is, preferred interactions)^11,12,21^. First, it is not clear how superlattice reflections can detect non-periodic SRO, or how scattering-based methods could detect instances of anti-clustering (non-preferred interactions). Second, a random atomic configuration of any alloy will inevitably contain local preferred solute interactions that can contribute diffraction effects. The diffracted intensity of these must be calibrated and separated from the experimental data to attain a measure of the net SRO. All of these represent significant challenges for the field.

**Fig. 1:**
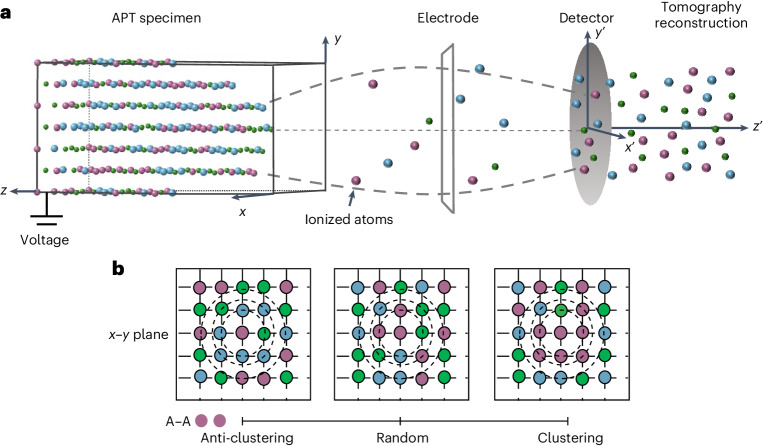
Schematic of APT and atomic behaviours. **a**, APT where the ionization of atoms under a standing field result in their field evaporation towards a detector. Time-of-flight measurements precisely indicate the mass-to-charge ratio, revealing the chemical identity of each individual evaporated ion. A position-sensitive detector enables a tomographic reconstruction atomic layer by atomic layer using a reverse-projection algorithm^29,31^. Millions (~10^6^–10^9^) of atoms from the analysed specimen can be reconstructed into tomographic atom maps^47^. The primacy of the issues of ionic trajectory and detector efficiency on the spatial resolution of the tomographic reconstruction is apparent. **b**, Two-dimensional schematic of anti-clustering, random and clustering SRO behaviours (refer to the purple atom pairs within first NN).

Atom probe tomography (APT) is a three-dimensional microscopy method where the surface atoms from a needle-shaped specimen under a positive electrical potential undergo ionization due to stimulation from either a high-voltage pulse or an ultrashort laser pulse. The technique is summarized in Fig. 1 and has had a significant impact on materials science and, relevant here, proven effective in characterizing solute–atom clustering, for example, in technologically important alloys^22,23^. Two key issues or challenges for using APT as a tool to measure SRO exist. The first is the challenge introduced by uncertainties in the true trajectories that the ions inevitably take as they detach from the specimen crystal via quantum mechanical interactions and take flight. Although the overall projection function describing how the average ion transits from the specimen to the plane of the detector is well researched^24–27^, uncertainties in the trajectory of any given individual ion remain. These uncertainties diminish the spatial resolution of the technique. The second is the fact that the detectors in APT have finite efficiency, presenting a classic missing data problem^28^. For example, ~43% of atoms^27,29^ in instrument systems such as that used in our work are not detected. Second-order issues also present, such as the need for a careful calibration of the reconstruction, as well as a careful calibration and ranging of the mass spectrum. However, some progress has been made on these issues, and techniques are available to mitigate them in a range of circumstances^27,29^. Here our focus is on the controversial question as to whether we can reliably and accurately measure SRO in these multicomponent alloys. We do this by addressing both key primary issues identified above using a data science approach. We quantify the SRO in a CoCrNi medium-entropy alloy (MEA) under different heat treatment conditions, unequivocally showing that the SRO can be engineered.

## Simulated SRO measurement

**Fig. 2:**
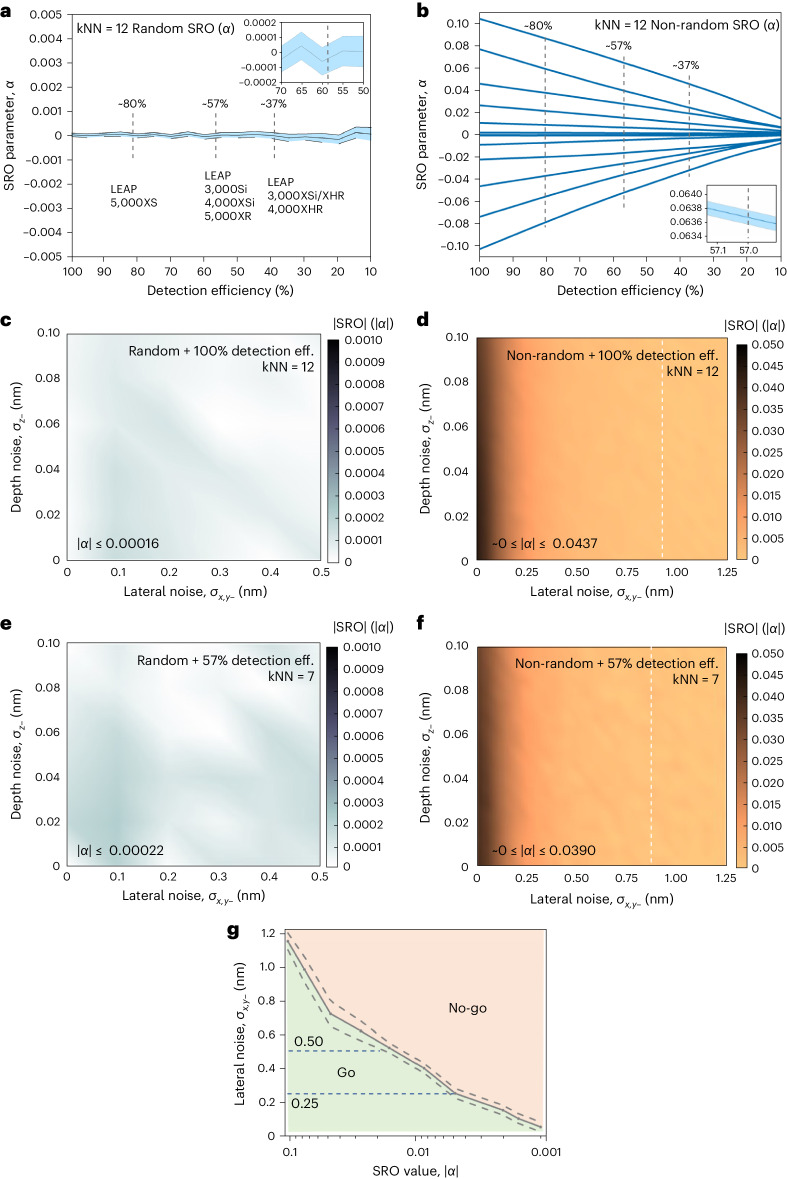
Understanding the influence of detector efficiency and spatial resolution. **a**,**b**, Influence of detection efficiency on the SRO parameter for random (**a**) and non-random (**b**) simulations. Averages and 95% confidence intervals are plotted from 100 simulations at each detection efficiency value. Annotations include the most common commercially available APT instruments. One example of the 95% confidence intervals is presented in the inset. The detailed maximum confidence intervals for **b** are presented in Supplementary Table 1. **c**,**d**, Influence of trajectory uncertainties (spatial resolution) on the SRO parameters at kNN = 12 for random (**c**) and non-random (**d**) simulations (arbitrary input, *α* = 0.041). **e**,**f**, Combined influence of 57% detection efficiency and trajectory uncertainties on the random (**e**) and non-random (**f**) cases (arbitrary input, *α* = 0.039) at kNN = 7. The SRO values are small across **c** and **e**, with the background colour near clear (white). The depth and lateral resolutions represent the standard deviation of Gaussian noise applied across the *x*–*y* (lateral) plane and *z* (depth) direction. The absolute values of the SRO are presented. The white dashed line indicates a contour where |*α*| ≤ 0.00022, the SRO threshold below which it is impossible to distinguish between random and non-random situations. This contour occurs at a lateral noise value of 0.95 nm in **d** and 0.85 nm in **f**. These tests have been repeated 100 times with similar trends. The standard deviation of these tests is plotted in Supplementary Fig. 1. **g**, Go/no-go threshold, with the standard deviation indicated by dashed lines, is presented on a logarithmic scale using SRO values ranging from ~0.100 to ~0.001 and *x*, *y* spatial noise. Below certain *x*, *y* spatial-noise levels, the SRO values are difficult to distinguish from random (|*α*| ≤ 0.00022). Source data

To evaluate the influence of detection loss and trajectory uncertainties^30,31^ on the measurement of the SRO value (*α*), a computational model of a face-centred cubic (fcc) CoCrNi MEA comprising ~4 million atoms was created. Two model systems were generated such that the atomic sites were assigned to the Co, Cr or Ni species by (1) random assignment (*α* = 0) and (2) non-random assignments including certain pairwise interactions that were clustered (*α* > 0), and other pairwise interactions that were anti-clustered (*α* < 0) (refs. ^32–34^) (Fig. 1b). The specific values of these imposed SRO parameters are summarized in Supplementary Table 1 and the computational technique for implementing these SRO values in the model system was the reverse Monte Carlo approach^35^. The influence of the APT detector efficiency was evaluated first by removing a proportion of the model atoms ranging from 0% to 90% by random selection and re-calculating the SRO of the system. Increments of 5% detection efficiency were used and for each value, the above process was repeated 100 times to build a statistical model. The algorithm for computing the SRO value is described in Methods and was the same for both simulation and experimental data throughout this paper. Figure 2a presents the average of 100 simulations and the corresponding 95% confidence intervals of the randomly assigned system at each value of detection efficiency sampled. The SRO values oscillate at around *α* = 0 across the range of detector efficiencies. The vertical dashed lines in Fig. 2a,b represent various typical APT instruments in use around the world, and their approximate detection efficiencies. Figure 2a (inset) shows the SRO values simulated near the detection efficiency of 57% (the value of our instrument^27,29^). At the 95% level of confidence, we find that for this detection efficiency, values |*α*| ≤ 0.00022 must be considered random. Figure 2b presents the results for the non-random system, where the specific values of the ‘true SRO’ embedded in the model for the different pairwise permutations are given at the intercepts to the ordinate axis, corresponding to 100% detection efficiency (the values are provided in Supplementary Table 1). Figure 2b demonstrates that all the SRO values tend towards zero almost linearly with an increasing fraction of missing atoms. Nonetheless, the models preserve the detection of deviation from a random distribution (that is, *α* ≠ 0) even when the fraction of missing data is high (>50%). Significantly, discerning whether SRO exists in a sample (*α* ≠ 0) and the capacity to follow the relative trend of the SRO values were retained. The measurement precision was clearly lost compared with the true values embedded in the simulation, with a systematic underestimation observed. The simulations based on an equiatomic alloy here indicate that the measured value of SRO derived from APT instruments with limited detector efficiencies will underestimate the true value by a discrete but determinable amount.

To assess the effect of trajectory uncertainties on atomic preferencing, we added Gaussian noise to the idealized atomic positions of the atomistic models^36^. Recognizing the well-known anisotropy in the spatial resolution of APT, we applied different noise regimes to the *x*–*y* plane, where the trajectory uncertainties are more evident, to that in the *z* direction (Fig. 1a), where the spatial resolution is better^37^. Figure 2c,d provides the results of the measured |*α*| values for SRO mapped in a coordinate space defined by the spatial resolution in the lateral and in-depth directions. Here the spatial resolution was quantified as the standard deviation of the Gaussian noise filter; therefore, the origin point of these charts represents an ideal microscope with no uncertainty in the trajectory, resulting in atomic positions precisely as per those generated in the input model. We have selected ranges for the spatial resolution in the lateral and in-depth directions that correspond to values typical of the estimations provided in the literature and that accord with our experience^38,39^. Calculation increments were 0.10 nm for lateral noise and 0.02 nm for in-depth noise. For the randomly assigned system (Fig. 2c), the SRO values fluctuate around 0 throughout the sampled range of spatial noise with |*α*| ≤ 0.00016. Figure 2d summarizes the results for the model generated with clear SRO using non-zero inputs for the *α* values. Specifically, this chart maps the measured |*α*| values for the Co–Ni pair and assigns an arbitrary pairwise SRO value of *α*_True_ = 0.041 at kNN = 12 (the coordination number for an fcc lattice). We find that this and all the |*α*| values investigated decrease towards 0 with increasing lateral spatial noise. These simulations are evidence that the measured SRO is predominantly influenced by spatial noise, particularly by the lateral spatial noise. Notwithstanding this, it remains the case that non-random values were detected even at high levels of spatial noise, with the simulated range of SRO spanning 0.0000013 ≤ *α* ≤ 0.0437000 for the noise parameter space, where *α*_True_ = 0.0410000. A contour is mapped in Fig. 2d (white dotted line) representing the conservative (larger) threshold value of |*α*| = 0.00022 (Fig. 2a) as the threshold at which random and non-random values are indistinguishable.

Next, atomistic simulations were generated to assess the combined effect of both finite detection efficiency (set here at 57%) and trajectory uncertainties on the measured SRO values. Results for the model containing random assignments of the Co, Cr and Ni species are charted in Fig. 2e, where the SRO values remain close to 0 throughout, at |*α*| ≤ 0.00022. Figure 2f charts the results from the same model as that in Fig. 2d, where the SRO for the Co–Ni pair was used at kNN = 7 to account for detection efficiency (since 0.57 × 12 ≈ 7), in which case the non-random value of *α*_True_ is 0.039. Inspection of the origin point (Fig. 2d,f) reveals the effect of the 43% missing data without any diminishment in spatial resolution. Here, in Fig. 2f, the SRO value was similar to the embedded *α*_True_ value (0.041) used in Fig. 2d where kNN = 12 and was 0.039 (since kNN = 7). The diminished spatial resolution in the lateral and in-depth directions drive the measured SRO values further down, with the lateral resolution again having the most acute impact. Non-random values of SRO were detected even at high levels of spatial noise, with the recorded SRO ranging within 0.0000086 ≤ *α* ≤ 0.0390000 for the simulated noise parameter space. The simulation in Fig. 2f is the most realistic and offers a potential map of the go/no-go region for which SRO values could be reasonably measured. Using the threshold value of *α* = 0.00022, non-random values of SRO may be discerned to the left of the white dotted line, requiring instrumental performance where the lateral noise was <0.85 nm. We proceeded to calculate this minimum lateral noise required for a range of SRO values (*α*_True_) between ~0.1 and 0 at 57% detection efficiency and the results are plotted in Fig. 2g. A go/no-go region is mapped for different SRO values and *x*–*y* (lateral) spatial noise. As the true SRO values (*α*_True_) decrease, the lateral spatial noise threshold for discerning non-random values also reduces—in other words, the instrumental performance thresholds become more demanding. Interestingly, the combined effect of spatial noise and detection loss does not artificially induce enhancements in the measure of the state of clustering or anti-clustering. Rather, they drive *α* → 0. Moreover, we find this diminishment in the SRO value to 0 to be a systematically linear relationship with both fraction of data loss and trajectory uncertainties. This monotonic relationship establishes a pathway to extrapolate back to the true SRO value in the material via data simulations.

The above analysis of the equiatomic ternary alloy finds embedded relationships between the monotonic diminishment of *α* → 0 as the spatial resolution and detector efficiency degrade. Next, we present an approach to reconstitute the true value of SRO from the (diminished) values measured experimentally, when it is possible to assign values for the spatial resolution and detector efficiency.

**Fig. 3:**
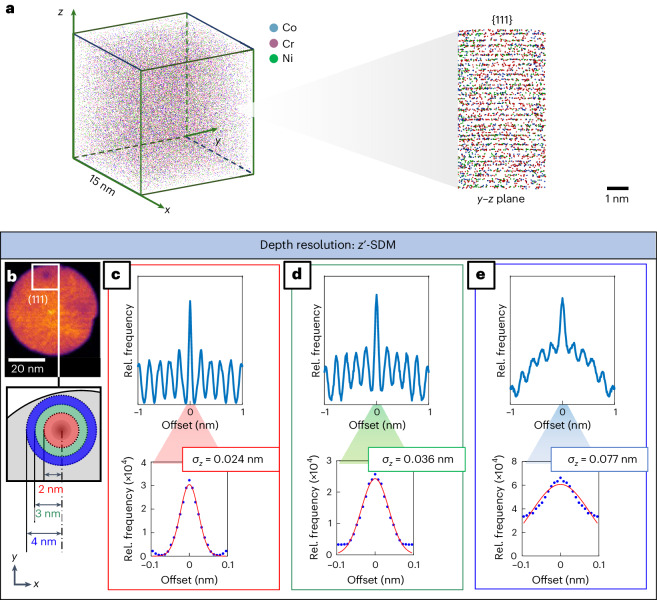
Reconstructed atom map and resolution assessment for APT. **a**, APT atom map from the AN500 sample, calibrated at the (111) pole; the inset confirms sufficient resolution to visually discern the {111} planes. **b**–**e**, Determination of in-depth resolution parameters. **b**, Two-dimensional density map from the CoCrNi MEA (AN500 condition) experimental APT reconstruction across the *x*–*y* plane—a (111) pole is observed. The diagram below shows the annular regions of interest in red, green and blue from which the in-depth spatial resolution was measured in the corresponding reconstruction. Panels **c**–**e** show the SDMs for the three regions at different distances from the (111) pole shown in **b**.

Our CoCrNi ingot was arc melted and homogenized at 1,200 °C for 24 h (the AH sample). The ingot was cut in half and annealed at 500 °C for 500 h (the AN500 sample). Further details are provided in Methods. Figure 3a shows an atom map prepared after calibrating the APT reconstruction from the AN500 sample and includes a zoomed-in image of the reconstructed {111} atomic planes along the *z* axis. Figure 3b provides a two-dimensional density map taken of the CoCrNi MEA (AN500 condition) experimental APT reconstruction across the *x*–*y* plane. A lower-density pole region corresponding to (111) is observed. The diagram below shows the annular regions of interest used to measure the in-depth spatial resolution in the corresponding reconstruction. The in-depth or *z*′ resolution across this region was estimated using spatial distribution maps (SDMs)^40^ generated from the annular regions close to the centre of the (111) pole. The Gaussian noise values (${\sigma }_{z-}$) were determined to be 0.024, 0.036 and 0.077 nm at annular regions of 0–2, 2–3 and 3–4 nm from the centre of the (111) pole, respectively (Fig. 3c–e). On this basis, ${\sigma }_{z^-}$ = 0.1 nm was selected for the in-depth resolution for subsequent simulations, consistent with what would be expected based on previous studies on APT resolution^41^. The lateral or *x*–*y* resolution was estimated using values reported in the literature^38,39^ at ${\sigma }_{x,y-}$ = 0.25 nm. The second and third examples of arbitrary true SRO values with an in-depth resolution of ${\sigma }_{z-}$ = 0.1 nm and a lateral resolution of ${\sigma }_{x,y-}$ = 0.5 nm (ref. ^42^) (Supplementary Fig. 2) and a lateral resolution of ${\sigma }_{x,y-}$ = 1.0 nm (Supplementary Fig. 3) are provided.

**Fig. 4:**
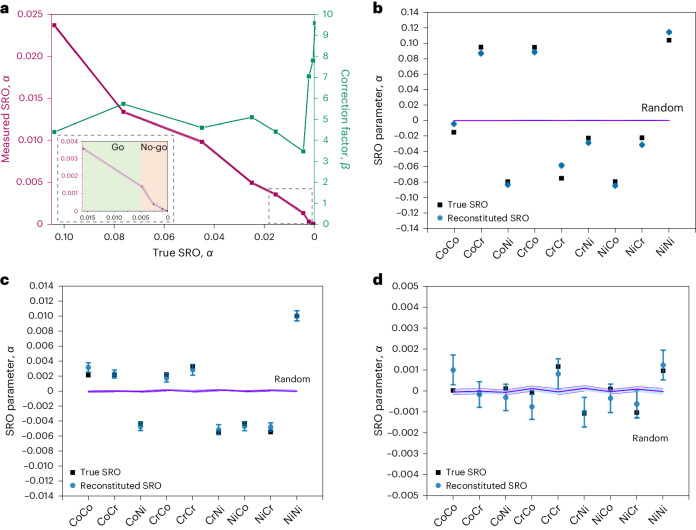
A reconstitution process to determine the true SRO. **a**, Measured SRO values (red) versus true SRO values (Supplementary Table 2) embedded in the simulated model of a CoCrNi MEA. This enables the determination of a correction factor *β*, which accounts for the combined effects of detection loss (57% detection rate) and limited spatial resolution (${\sigma }_{x,y-}$ = 0.25 nm, ${\sigma }_{z-}$ = 0.10 nm) for each value of SRO for this alloy system (green). The low-SRO range shown in the inset and Fig. 2g was used to determine the go/no-go threshold value for SRO below which random and non-random events are indistinguishable (that is, *α* < 0.0048). **b**–**d**, Validation of the reconstitution procedure via a comparison between (arbitrary) true SRO values (black) to the reconstituted SRO values (blue). Three sets of arbitrary true values were assessed to test high (**b**), medium (**c**) and low (**d**) SRO values (the values are listed in Supplementary Table 3). The data points (blue) show the 95% confidence intervals. 57% of the data was simulated 100 times using the random labelling method with ~4 million atoms, and the SRO was measured for kNN = 7 to generate the range labelled random (violet). Data are presented as the average of the reconstituted SRO value for each pair ± their 95% confidence region. The SRO values for random simulations ranged for |*α*| ≤ 0.00022. Source data

New CoCrNi MEA atomistic models containing ~4 million atoms were generated. Various pairwise SRO values ranging from ~0.1 to 0 were assigned to the nine possible different pairwise permutations, (Fig. 4a and Supplementary Table 2). The resultant atomistic model was then subjected to a random removal of 43% of the atoms, and a random Gaussian noise of standard deviation ${\sigma }_{x,y-}$ = 0.25 nm (laterally) and ${\sigma }_{z-}$ = 0.10 nm (in depth).

The pairwise SRO values were then re-measured and recorded and the process was repeated 100 times. The results are charted in Fig. 4a, where the red curve directly compares the measured versus the true *α* values for this simulation regimen. A correction factor *β* is also charted in green on the alternate ordinate axis (Fig. 4a). This was determined by dividing the assigned true SRO value by the measured SRO value after the degradations from detection efficiency and trajectory uncertainties were applied to the initial atomistic model:

1 $${{\beta }}={{\rm{\alpha }}}_{{\rm{True}}}/{{\rm{\alpha }}}_{{\rm{Measured}}}.$$

This correction factor was separated into two regimes: a sharp tendency for this quotient for *β* → ~10 when there was little or no SRO, and a flat region where there exists a medium level of SRO, such that for *α* > 0.0014, ~3.7 ≤ *β* ≤ ~5.7. The low-SRO range is enlarged in Fig. 4a (inset). Using the values calculated in Fig. 2g, we see that it is impossible to distinguish between SRO values for *α*_True_ < 0.0048 when the spatial noise is 0.25 nm, and hence, the go/no-go threshold is mapped accordingly.

These correction factors were validated using models embedded with three sets of arbitrary true SRO values that had 57% detector efficiency and values of spatial noise (0.25 nm laterally; 0.10 nm in depth) applied 100 times. The three sets of input true SRO values are tabulated in Supplementary Table 3 and notionally correspond to high SRO (~0.0100 < *α* < 0.1000; Fig. 4b), medium SRO (~0.0010 < *α* < 0.0100; Fig. 4c) and low SRO (~0.0001 < *α* < 0.0010; Fig. 4d). The correction factors (*β*) determined from Fig. 4a enable a system of equations to be used on the measured SRO values to reconstitute the true SRO values, thereby accounting for the monotonic degradations arising from the finite detection efficiency and trajectory uncertainty. Using the threshold of |*α*| ≤ 0.00022 for the measured SRO values that must be considered indistinguishable from random, it is demonstrated (Fig. 4) that an APT with a detector efficiency of ≥57% and spatial noise within the thresholds used here will unequivocally return acceptable precision for high and medium input levels of SRO (Fig. 4b,c) but not for low input levels of SRO (Fig. 4d). In Supplementary Figs. 2 and 3, we repeat this same simulation process for higher levels of lateral spatial noise. The standard deviation (${\sigma }_{x,y-}$) of the random Gaussian noise was set to 0.50 and 1.00 nm (Supplementary Figs. 2 and 3, respectively). The trend is that the minimum threshold value of SRO that can be detected is higher as the lateral noise increases.

## Experimental SRO measurement

**Extended Data Fig. 1:**
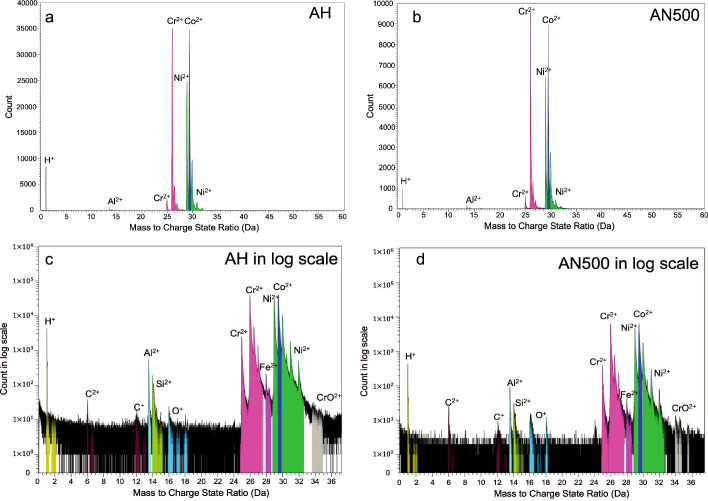
Mass-to-Charge ratio spectra from APT experiments. (**a**) The mass spectrum for the AH condition, showing peaks for the constituent elements H, Al, Cr, Co, and Ni. (**b**) The mass spectrum for the AN500 condition, with corresponding elemental peaks. The spectra illustrate the relative intensities of detected ions, with negligible presence of H, C, N, O, Al, Fe and Si and prominent peaks for the Co, Cr and Ni elements. (**c**) and (**d**) show the enlarged log scale to the (**a**) and (**b**).

As shown in Fig. 3a, the crystallographic planes were clearly resolved in our reconstructed APT data of the MEA, and the atomic mass-to-charge ratios in the MEAs enable a total differentiation between the Co, Cr and Ni species without any overlap in the mass spectrum (Extended Data Fig. 1). With these secondary considerations accounted for, the primary issues of spatial resolution and detector efficiency remain. We applied the reconstitution procedure described above.

**Fig. 5:**
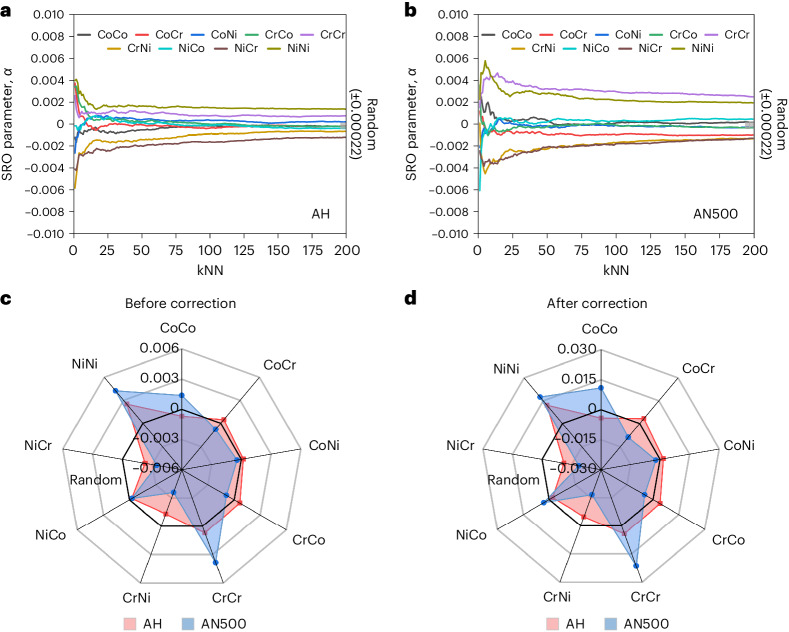
Comparison of SRO in AH and AN500 samples. **a**, Accumulated 1NN to 200NN SRO calculations of the entire APT dataset of AH (**a**) and AN500 (**b**). In the AH sample, all the pairs exhibited SRO values in the range of −0.00112 ≤ *α* ≤ 0.00141 at 200NN, indicative of little or no SRO, effectively showing a near-random distribution. In the AN500 sample, the preferencing between the atomic species was more pronounced, with the pairwise SRO values ranging within −0.00602 ≤ *α* ≤ 0.00557. The Ni–Ni and Cr–Cr pairs tended to cluster, whereas the Ni–Cr pairs exhibited anti-clustering. After annealing, the degree of clustering of Cr increased, with Cr–Cr values changing from 0.00080 in the AH condition to 0.00409 in the AN500 condition. The tendency for anti-clustering of the Cr–Ni increased after annealing, from −0.00063 in the AH state to −0.00453 in the AN500 condition. The random regions (±0.00022) were shown in grey in both figures. The NN–distance relationships in an ideal CoCrNi model are 0.25 nm for 1NN–12NN, 0.36 nm for 12NN–18NN and 0.44 nm for 18NN–42NN. **c**,**d**, Comparison of SRO before (**c**) and after (**d**) reconstitution for different atom pairs. These results were obtained by averaging two of our experimental APT datasets for each condition, with a focus on the seventh nearest neighbour (kNN = 7). The data were extrapolated to account for no noise and 100% detector efficiency, using the corrections and simulations developed in this study. These corrections and simulations assume a lateral resolution of 0.25–0.50 nm and an in-depth resolution of 0.10–0.20 nm. The standard deviation of error associated with these estimates is detailed in Supplementary Table 4. Source data

**Extended Data Fig. 2:**
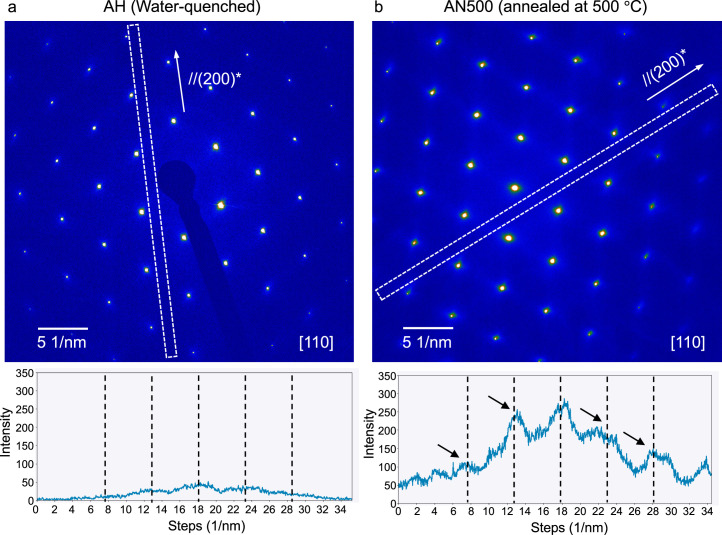
TEM energy filtered diffraction patterns and intensity analysis. Panel (a) displays the [110] orientation diffraction pattern for the AH sample with the intensity profile extracted from the rectangular region outlined, showing uniform intensity without significant fluctuations. Panel (b) shows the [110] orientation diffraction pattern for the AN500 sample with the intensity profile from the rectangular region outlined, where the peaks indicated by arrows reveal periodic intensity variations, indicative of the presence of SRO. The rectangular regions in both (**a**) and (**b**) are aligned parallel to the (200)* planes.

**Extended Data Fig. 3:**
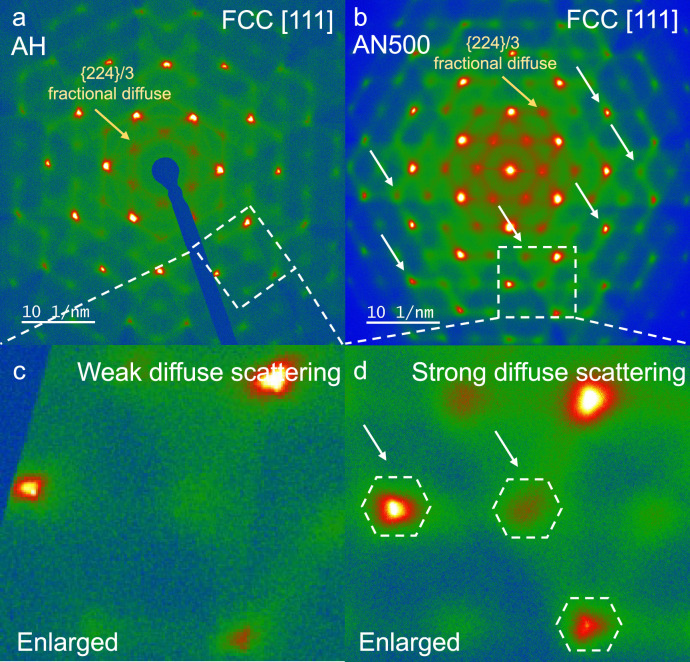
Comparative TEM diffraction patterns between AH and AN500 from [111] orientation. (**a**) shows the FCC [111] diffraction pattern from the AH sample, with weak diffuse scattering evident, and (**c**) provides an enlarged view highlighting this feature around the bright dots. (**b**) depicts the FCC [111] diffraction pattern for the AN500 sample, with strong diffuse scattering observed, which is further emphasized in the enlarged view in (**d**). The fractional diffuse scattering in (**a**) and (**b**) is marked by the {224}/3 planes indicated by yellow arrows. The white arrows and dashed circles in (**d**) indicate regions of pronounced diffuse intensity, characteristic of SRO.

**Extended Data Fig. 4:**
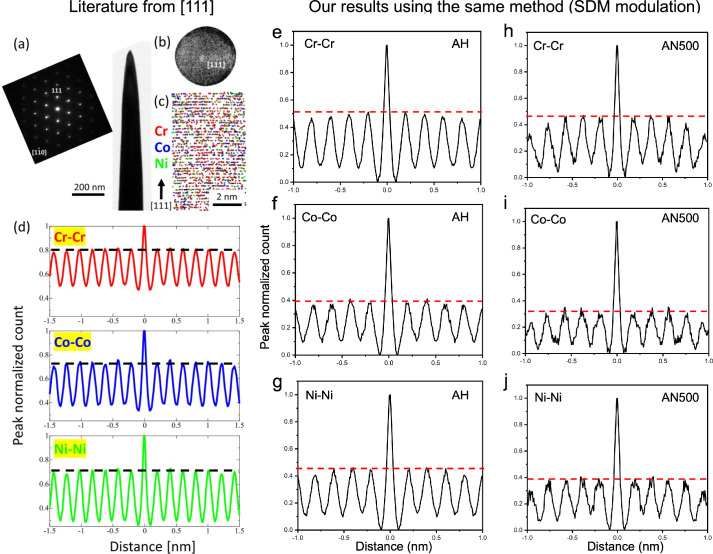
Comparative Analysis: Results from the literature versus our tests using APT-SDM aligned along the [111] axis. (**a**) TEM image and diffraction pattern of the needle specimen fabricated so that the [111] direction coincides with the needle axis in the annealed sample. (**b**) 2D desorption image showing central pole [111]. (**c**) Atom map slice (5-nm thick) enlarged in the central pole region. (**d**) SDM of Cr-Cr, Co-Co, and Ni-Ni along the [111] direction^15^. (**e**–**g**) present our AH sample results using the same SDM method, with peak correlations for Cr-Cr, Co-Co, and Ni-Ni pairs. (**h**–**j**) depict the AN500 sample results. The black dashed lines and red dashed lines in the figures use the first peak as a reference to show the amplitude of the peaks.

**Extended Data Fig. 5:**
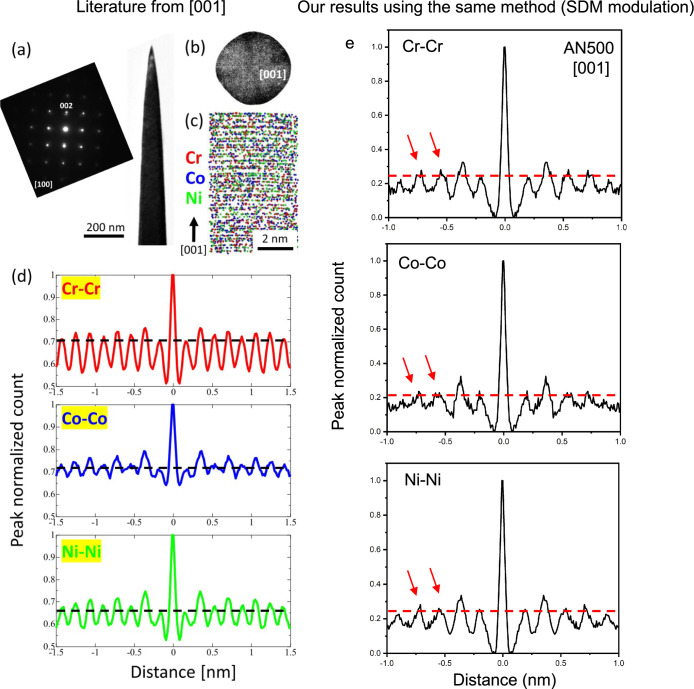
Comparative Analysis: Results from the literature versus our tests using APT-SDM aligned along the [001] axis. (**a**) TEM image and diffraction pattern of the needle specimen fabricated so that the [001] direction coincides with the needle axis in the annealed sample. (**b**) 2D image shows central pole [001] and zone lines, having a fourfold symmetry. (**c**) Atom map slice (5-nm thick) enlarged in the central pole region. (**d**) SDM of Cr-Cr, Co-Co, and Ni-Ni along the [001] direction.^15^ (**e**) illustrates our results using the same SDM modulation method for the AN500 sample in the [001] orientation, displaying the peak-normalized count for Cr-Cr, Co-Co, and Ni-Ni pairs. The red arrows indicate significant deviations from randomness, suggesting the presence of SRO as characterized by peak correlations at certain atomic distances.

Figure 5a,b presents the results of calculating the cumulative SRO parameter from the first nearest neighbour (1NN) to the 200th nearest neighbour (200NN) of the CoCrNi MEAs in the AH and AN500 conditions by using the entire dataset with at least 2 million atoms. Each SRO calculation considers all the atoms in a sphere out to the respective kNN value. After ~50NN, all the pairwise SRO values in both conditions converged to a steady state, and the value at 200NN was used as the experimental SRO value between the different pairs (as shown). This result indicates that annealing has driven a clear departure from the random distribution apparent in the AH condition, with significant changes particularly for the Cr–Cr and Cr–Ni/Ni–Cr pairs. These experimentally measured SRO values were then used as inputs to the reconstitution algorithm described above. Before and after the reconstituted values are presented in Fig. 5c,d for both AH and AN500 samples of the CoCrNi MEA. The existence and conditions of SRO in AN500 and the near-random condition of the AH sample have been further confirmed using transmission electron microscopy (TEM) (Extended Data Figs. 2 and 3)^11,43^ and APT spatial distribution methods (Extended Data Figs. 4 and 5)^15^ following published methods.

## Conclusions

Our work suggests a promising outlook for generating realistic atomistic models based on experimental APT data. This is significant because these models can then serve as the starting point for computational materials simulations using density functional theory and/or molecular dynamics^44^ (Supplementary Discussion 1). The method for the determination of SRO introduced here might be described as being data driven^35^, and it is interesting to compare this with the recently published approach based on image processing^45,46^. Both methods iterate between data generated via simulations and experiments. Our approach applies a data science technique to map the relationships between SRO values, detector efficiency and spatial noise in APT data (Supplementary Discussion 2).

This study has demonstrated a reconstitution procedure that serves as a method to quantitatively measure the SRO in an equiatomic CoCrNi MEA (Supplementary Discussion 3). When applied to homogenized CoCrNi samples (AH) and samples annealed at 500 °C for 500 h (AN500), clear changes in the SRO for the various elemental pairs were apparent (Fig. 5). Cr–Cr and Ni–Ni pairs were found to cluster, whereas Cr–Ni and Ni–Cr pairs were found to exhibit anti-clustering. These findings underpin a framework that is both alloy specific and instrument specific, facilitating the delivery of real-world atomistic data to computational simulations to further explore microstructural evolution and materials properties.

## Methods

### Materials and processing

The CoCrNi alloy was arc melted using equal molar fractions of high-purity Co, Cr and Ni in an argon atmosphere. The as-cast alloy was then homogenized at 1,200 °C for 24 h followed by water quenching, and this sample was designated AH. A small piece of the AH alloy was cut and measured using inductively coupled plasma atomic emission spectroscopy to obtain a statistical average of the alloy molar fraction, which confirmed near equiatomic ratios of the constituent species (Supplementary Table 5). Based on earlier literature regarding the CoCrNi alloy^14^, 500 °C was chosen as the temperature to induce SRO. Therefore, half of the AH alloy was cut and placed in a salt bath at 500 °C annealing temperature for 500 h followed by a water quench (the sample prepared under this condition is named AN500).

Samples for the X-ray diffraction (XRD) and scanning electron microscopy (SEM) tests were cut into small pieces using Struers 50 diamond saw and then polished using silicon carbide sanding papers up to 4,000 grit followed by electropolishing. The samples were polished to mirror-like surfaces. The electropolishing was conducted using 10% perchloric acid in acetic acid at room temperature.

### Microstructure of the samples

The XRD was analysed using a STOE STADI P X-ray diffractometer configured with a molybdenum (Mo) source. The samples were tested from 15° to 50°. The electron backscatter diffraction detector (EBSD) and electron diffraction X-ray spectrometry were done using a Zeiss Ultra SEM instrument equipped with electron diffraction X-ray spectrometry and EBSD.

Both AH and AN500 alloys were measured to have single fcc using XRD and EBSD. The phase and lattice parameter for both samples were confirmed to be similar (Supplementary Fig. 4). No obvious differences in atomic local concentration distribution (Supplementary Fig. 5) were found between the two samples.

### APT sample preparation and experiments

At least two APT specimens of each condition (that is, AH and AN500) were prepared using the lift-out method on a Thermo Fisher G4 Hydra Plasma focused-ion-beam SEM instrument equipped with an Oxford Instruments EBSD detector. The same crystal direction, namely, [110], was arbitrarily chosen to be aligned to the *z* axis or the length of the tip specimen from both conditions by using correlative EBSD maps collected before lift-out and selecting grains with the same inverse pole figure *z* orientation. Annular milling was used on the lift-out samples to reach a radius of less than 100 nm APT tips. To evaluate the effects of pole orientations, studies have been conducted on both [100] and [111] orientations under varying conditions. The results of these studies are presented in Extended Data Fig. 7.

APT experiments were carried out using a CAMECA LEAP 3/4000Si instrument. The running condition for the samples was 20.0% pulse fraction, 200 kHz pulse rate, 50 K and detection rate of 0.2% (ref. ^48^). The data were reconstructed and analysed using the Interactive Visualization and Analysis Software package (IVAS 3.8.4 and AP Suite 6.1) to achieve the calibrated position and mass-to-charge ratio of each species in three-dimensional space (Extended Data Fig. 1). Extended Data Fig. 1 demonstrates that the content of impurity elements C, N, O, Fe, Al and Si are negligible. They do not substantially change between the AH and AN500 states and therefore do not significantly influence the SRO measurement. H is detectable in both conditions; most of the H is attributed to background H from the chamber^49^ and not from the material itself; hence, it has no influence on the SRO. Inductively coupled plasma atomic emission spectroscopy (Supplementary Table 5) also found that impurity elements accounted for only 0.26% in the original CoCrNi ingot.

**Extended Data Fig. 6:**
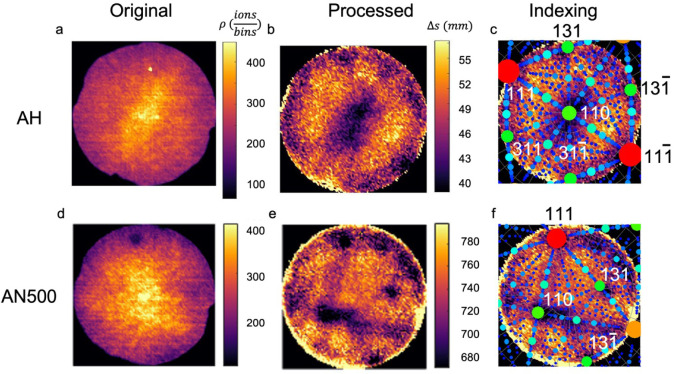
Density hit maps and pole identification process for AH and AN500 heat treatment conditions. (**a**, **d**) original hit maps, (**b**, **e**) processed hit maps mapping average distance between sequential detector events per pixel, and (**c**, **f**) indexed poles from the processed figures. Multiple poles are clearly identified from the figures for the reconstruction process.

After processing the APT data for both AH and AN500 conditions, the desorption maps were plotted. By using the density-mapping technique^50^ in combination with the known grain orientation, at least two poles were identified. ICF and *k*_f_ values were then calibrated for the APT data (Extended Data Fig. 6). Similar SRO trends were obtained from different tips for each condition.

### TEM sample preparation and experiments

Samples for TEM were sliced by a diamond saw (Struers 50) and thinned by mechanical polishing to 70 μm foils. The foils were punched to disks (diameter, 3 mm). Electron-transparent samples for TEM observation were prepared by twin-jet electropolishing that was carried out using a solution of 10% perchloric acid in methanal at –30 °C. The thin regions in the TEM specimen were used for the TEM experiments.

The TEM samples of different heat treatments were used for observation. A JEOL 2200 microscope (200 kV), equipped with an omega energy filter, was used to take both diffraction patterns. By using the energy filter, zero-loss peaks were selected to take the diffraction patterns. Gatan DigitalMicrograph 3.5 software was used to analyse the images.

### Pairwise SRO formalism

General theories for SRO stem from the Warren–Cowley treatment^32,51^ and include the derivative formalisms of PM-SRO^33^ and GM-SRO^34^. The SRO parameter used in this study is similarly based, and our derivations are provided. SRO is expressed as a dimensionless parameter referred as *α*, such that −1 ≤ *α* ≤ 1. When the atomic species under consideration are distributed randomly, *α* = 0. When these species are clustered together due to preferred interactions, then *α* > 0; when anti-clustering occurs, these species tend to be repelled and *α* < 0 (Fig. 1b).

The Warren–Cowley SRO formalism was originally derived for a dilute binary alloy system. To cover the more complicated situation that arises in M/HEAs with compositional complexity, we have modified the expression for the SRO parameter *α*. The purpose of this revision was to simplify the mathematics for the M/HEA-type cases. The revised pairwise SRO parameter used here was ${\alpha }_{{{\rm{AB}}}}^{m}$ as defined by the following equation:

2 $${\alpha }_{{{\rm{AB}}}}^{m}=\,\frac{{P}_{{{\rm{AB}}}}-{X}_{{\rm{B}}}}{{X}_{{\rm{B}}}}.$$

Here *α* is the SRO parameter, *m* is the shell, *X*_B_ is the concentration of B atoms calculated from the entire atom probe dataset and *P*_AB_ is the probability of finding atom B around a central atom A within certain NN atom amounts. For the system examined here, A and B correspond to the pairwise instances of the Co, Cr and Ni atomic species. Supplementary Table 6 demonstrates the relationship between this definition of ${\alpha }_{{{\rm{AB}}}}^{m}$ and the definitions used in other work; moreover, it demonstrates that the expressions for SRO are equivalent.

### Simulation of SRO

Step 1: build the model. A perfect fcc lattice containing ~4 million lattice sites with lattice parameter *a* = 0.36 nm was generated. The lattice sites were randomly occupied by Co, Cr and Ni atoms in equal concentration. This simulation was the basis for the *α* = 0 random case. The atomic positions were randomized 100 times using Monte Carlo simulations published on the CVL platform^35^. The crystal structure and lattice parameters were assigned based on the XRD and EBSD experiment results (Supplementary Fig. 4). The simulation was generated using MATLAB R2020.

Step 2: detector efficiency simulation. The three elements were randomly distributed in the model using a random labelling method (Fig. 2a). Additionally, SRO-enriched models were built by forcing the atomic preference within kNN = 12 to various pairwise SRO values (0.1 to −0.1) within the extreme conditions for MEAs using reverse Monte Carlo simulations^35^ (Fig. 2b). The influence of the detection efficiency on the SRO parameter for random and non-random simulations (Fig. 2a,b) was simulated from 100% detection efficiency to 10% detection efficiency with a step size of −5%. Each point was simulated 100 times, and average values and 95% confidence level are plotted in Fig. 2a,b.

Step 3: spatial noise simulation. Figure 2c,d simulates the use of fixed SRO value pairs. For the random case, one random labelling model with a fixed random pair was used (Fig. 2c). The lateral noise was simulated from 0 to 0.5 nm with a step size of 0.1 nm. The depth noise was from 0 to 0.10 nm with a step size of 0.02 nm. One hundred simulations were done, and the absolute value of the average SRO values was plotted. For the non-random case, models with the same pair were chosen. An SRO value of 0.041 at kNN = 12 was used (Fig. 2d). In Fig. 2d, both lateral noise and depth noise were simulated.

This spatial noise is added by Gaussian noise characterized by a standard deviation (*σ*) with a mean of zero. We added lateral noise (that is, ${\sigma }_{x,y-}$) ranging from 0 to 1.25 nm and depth noise (that is, ${\sigma }_{z-}$) from 0 to 0.10 nm to mimic the real APT resolutions. For example, given the nature of Gaussian distribution, with a mean of 0 nm, a *σ* value of 0.25 nm implies that 68% of the noise falls within this range, but 95% of the noise added corresponds to 2*σ*, which is 0.50 nm. To maintain clarity, we report *σ* values instead of claiming 95% at 0.5 nm. Our method was tested under three conditions: (1) ${\sigma }_{x,y-}$ = 0.25 nm to $2{\sigma }_{x,y-}$ = 0.50 nm; (2) ${\sigma }_{x,y-}$ = 0.50 nm to $2{\sigma }_{x,y-}$ = 1.00 nm; (3) ${\sigma }_{x,y-}$ = 1.00 nm to $2{\sigma }_{x,y-}$ = 2.00 nm. For the first two conditions, our method remains effective (Fig. 4 and Supplementary Fig. 2), surpassing the standard material’s tested resolution of <0.28 nm to a worse condition of 1.00 nm (Supplementary Fig. 6).

Step 4: simulation of detector and spatial noise. Combining the effect of detector efficiency and spatial noise, the data in Fig. 2e,f were simulated using fixed SRO value pairs but various kNN numbers. For the random case, one random labelling model with a fixed random pair was used (Fig. 2e). In the beginning, 43% of the atoms are randomly removed. Then, the lateral noise was simulated from 0 to 0.5 nm with a step size of 0.1 nm. For the non-random case, models with the same pair were chosen. 43% random removal was applied, and an SRO value of 0.039 at kNN = 7 was used (Fig. 2f). The lateral noise was simulated from 0 to 1.25 nm. Random removals were done 100 times, and simulations were done; then, the absolute value of the average SRO values was plotted.

### Determination of spatial resolution

The spatial resolution of APT is instrument based, highly anisotropic and greatly dependent on the selection of experimental parameters and the material under analysis. We initially assessed our instrument using standard aluminium samples (Supplementary Fig. 6). The spatial resolution is higher in the in-depth direction than laterally by as much as an order of magnitude^42,52^. The in-depth resolution is the highest around the crystallographic pole features where the evaporation sequence is highly ordered, but these regions also have the lowest lateral resolution where local magnification effects brought about by trajectory uncertainties are more pronounced. The method described in another work^36^ was used to determine the representative values for spatial resolution for the collected experimental data.

Trajectory uncertainties were separated into two components: lateral resolution (*x*, *y*) and depth resolution (*z*). By considering the peak width of the central peak of a one-dimensional SDM generated along the normal direction to the {111} planes shown in Fig. 3b–e, the standard deviation of in-depth noise (${\sigma }_{z-}$) in this region was found to be 0.024, 0.036 and 0.077 nm. These represent the highest in-depth resolutions of the dataset. The in-depth resolution will degrade at regions further away from the (111) pole. A conservative value of ${\sigma }_{z-}$ = 0.1 nm for the in-depth resolution was used in the studies, consistent with existing literature on APT resolution studies including recent simulation studies^41^ that modelled the influence of changing evaporation fields in concentrated solid solutions or high-entropy alloys. Lateral resolution could not be directly measured as the spatial noise was too high to resolve crystallographic information from a two-dimensional SDM^53^. However, based on estimates from previous studies^41,42^, the lateral spatial noise is expected to have a standard deviation of approximately ${\sigma }_{x,y-}$ = 0.25 nm. If it were much higher, any SRO within the analysed dataset below *α* ≈ 0.0048 would not be detected (Fig. 2g). However, Fig. 5 clearly shows a measurable change in SRO between the experimentally derived AH and AN500 samples below these values.

### Simulation of APT experiments

In the SRO parameter calculations (Supplementary Table 6), since the signal of the SRO values was only representing the clustering/ordering (plus sign) and anti-clustering (negative sign) behaviours, the absolute value (|*P*_AB_ − *X*_B_|) is showing deviation from the concentration. Different true SRO values were embedded in the simulated models of a CoCrNi MEA. Gaussian noise with different standard deviation values (condition1: *x*, *y* = 0.25 nm and *z* = 0.10 nm; condition2: *x*, *y* = 0.50 nm, *z* = 0.10 nm; condition3: *x*, *y* = 1.00 nm, *z* = 0.10 nm) was added to the synthetic data and the SRO parameters were calculated. This enables the determination of a correction factor *β* using equation (1) (Fig. 4a and Supplementary Figs. 2a and 3a).

### Reconstitute and validate the SRO values

Nine pairs of a CoCrNi dataset (comprising 4 million atoms) with a perfect lattice was set to different true SRO values at kNN = 12 with 100% atoms. The APT simulation (43% detection loss; *x*, *y* = 0.25 nm, *x*, *y* = 0.50 nm; *x*, *y* = 1.00 nm; *z* = 0.10 nm noise) was applied to the dataset 100 times for each pair. The measured SRO values after the degradations were reconstituted with the correction factors to get a distribution with the mean value and 95% confidence intervals (Fig. 4b–d and Supplementary Figs. 2b–d and 3b).

### Experimental SRO calculation

The experimental SRO parameter was calculated using scripts run under MATLAB R2020. Based on equation (2), three parameters were used during the calculation.

First, *X*_B_ is calculated from the dataset for each species. The number of B atoms in the whole dataset is taken and divided by all the atoms in the dataset. *X*_B_ is calculated for all the three elements (Co, Cr and Ni).

Second, *P*_AB_, which is the probability of finding B around a central atom A within specific nearest atom amounts, is obtained. The nearest atom amounts (*m*) are set from 1 to 200 (Fig. 5a,b). Then, Co, Cr and Ni are selected as the central atom A and find the Co, Cr and Ni atoms around the central atom and calculate the probability.

Third, the pairwise SRO parameter is calculated after obtaining *P*_AB_ and *X*_B_. The numbers are saved and plotted as shown in Fig. 5a,b. Random labelling tests have also been conducted to prove the existence of SRO in the experimental results (Supplementary Fig. 7).

### Pole assessment and extraction process

#### Sample preparation and APT experiments

**Extended Data Fig. 7:**
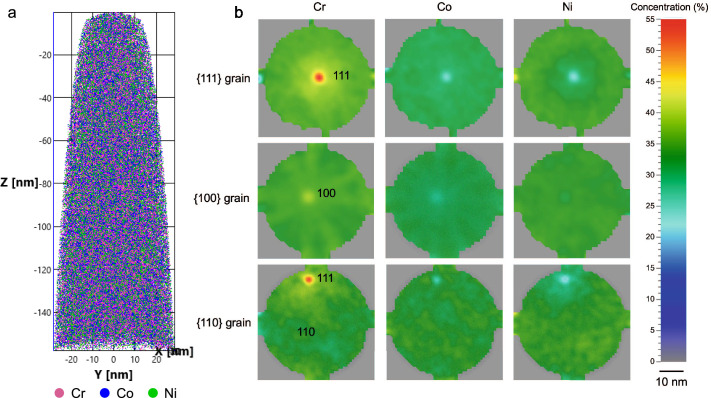
APT analysis of elemental distribution in different crystallographic orientations. (**a**) shows a three-dimensional reconstruction of an APT sample, with the atoms color-coded by element. (**b**) displays 2D concentration maps for Cr, Co, and Ni in three different grain orientations: {111}, {100}, and {110}. Each row corresponds to a different orientation, as indicated, with the concentration of each element represented in colour scale. These maps reveal the spatial variation of element concentration within the grains, providing insight into the distributional homogeneity across the different crystallographic orientations.

We prepared samples from the {111} and {100} orientations for both AH and AN500 conditions of CoCrNi, using consistent parameters and methods across all the orientations. The results (Extended Data Fig. 7) show varying concentration changes around the poles in different orientations, with the {111} orientation showing the most pronounced changes.

#### Pole influence on concentration

**Extended Data Fig. 8:**
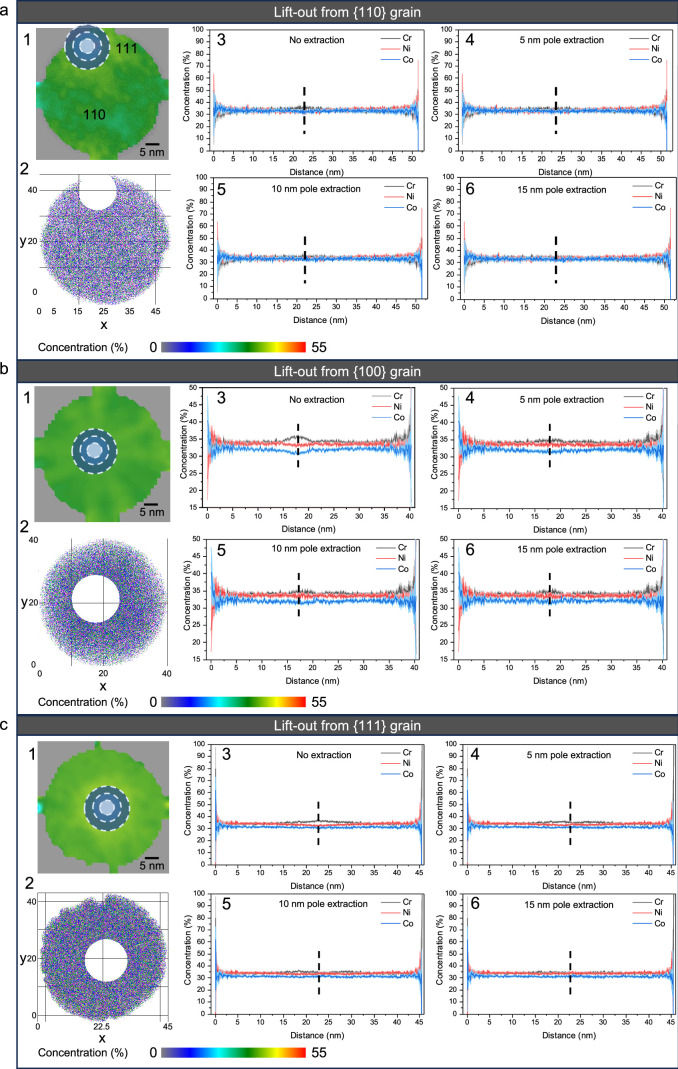
Analysis of elemental concentration variations by APT with pole extraction from different crystallographic orientations. (**a**) Lift-out from {110} grain. a1 depicts the APT concentration map with a {111} pole, and a2 shows the corresponding atomic map with pole extraction. a3-6 represent concentration profiles for Cr, Ni and Co at varying distances along x axis with no extraction, and following 5 nm, 10 nm, and 15 nm pole extraction, respectively. (**b**) Lift-out from {100} grain. b1 illustrates the APT concentration map with a {100} pole, and b2 shows the corresponding atomic map with pole extraction. b3-6 display concentration profiles for Cr, Ni and Co at varying distances along x axis with no extraction, and after 5 nm, 10 nm, and 15 nm pole extraction, respectively. (**c**) Lift-out from {111} grain. c1 demonstrates the APT concentration map with a {111} pole, and c2 provides the associated atomic map with pole extraction. c3-6 show concentration profiles for Cr, Ni and Co at different distances along x axis with no extraction, and following 5 nm, 10 nm, and 15 nm pole extraction, respectively. The concentration profiles highlight the influence of pole proximity and extraction size on the distribution of elements within the grains. The dashed line indicates the potential pole regions.

Extended Data Fig. 7b indicates that Cr is more abundant in the pole regions, whereas Ni/Co is less abundant. This is attributed to the differences in the evaporation fields of these elements. Cr has the lowest evaporation field at 29 V nm^–1^, whereas Co and Ni have higher fields at 37 and 36 V nm^–1^, respectively. The concentration fluctuation varying with orientation is shown in Extended Data Figs. 7 and 8: approximately 3 nm for the {110} direction (Extended Data Fig. 8a), around 5 nm for the {100} direction (Extended Data Fig. 8b) and about 7–8 nm for the {111} direction (Extended Data Fig. 8c).

Examining the impact of size and concentration on pole extraction (Extended Data Fig. 8): we studied the effect of different pole extraction sizes on concentration. After removing 10 nm in the {111} orientation, the concentration tended to be uniform, whereas a 5 nm extraction was sufficient for the {100} and {110} orientations. The {110} orientation’s primary influence was found to be the {111} pole but at the edge.

#### SRO results after pole extraction

**Extended Data Fig. 9:**
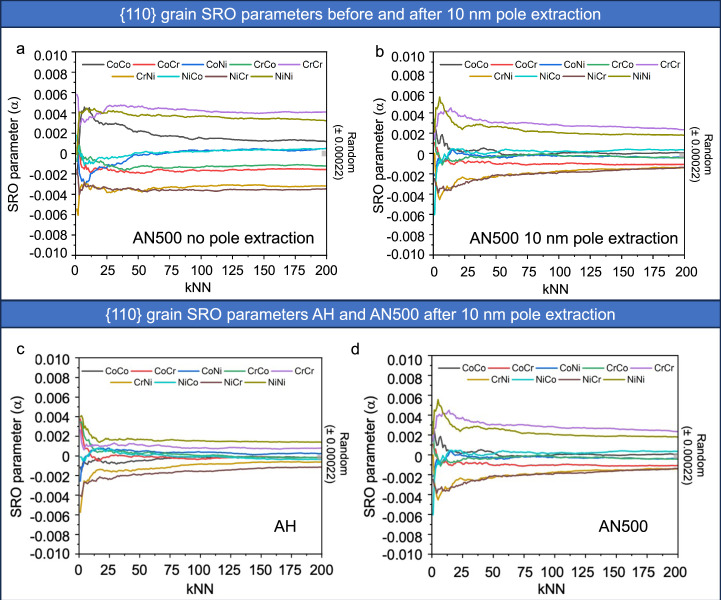
Analysis of SRO parameters in {110} grain orientation. (**a**) and (**b**) display the SRO parameters for the AN500 sample without pole extraction and after 10 nm pole extraction, respectively. (**c**) and (**d**) compare the {110} grain SRO parameters between the AH and AN500 samples following 10 nm pole extraction. Each graph plots the SRO parameter against kNN, illustrating the elemental pair correlations for Co-Co, Co-Cr, Co-Ni, Cr-Co, Cr-Cr, Cr-Ni, Ni-Co, Ni-Cr, and Ni-Ni.

In the {110} orientation, we compared the SRO results after extracting 10 nm with those without pole extraction (Extended Data Fig. 9a,b). The overall trend remained consistent, suggesting that preferential evaporation in APT, especially for the {110} grain, has a minimal impact after pole extraction. We conducted a comparative analysis of AH and AN500 following a 10 nm pole extraction (Extended Data Fig. 9c,d). This comparison reveals a marked difference in the SRO trends between the two samples.

#### Final pole extraction process

**Extended Data Fig. 10:**
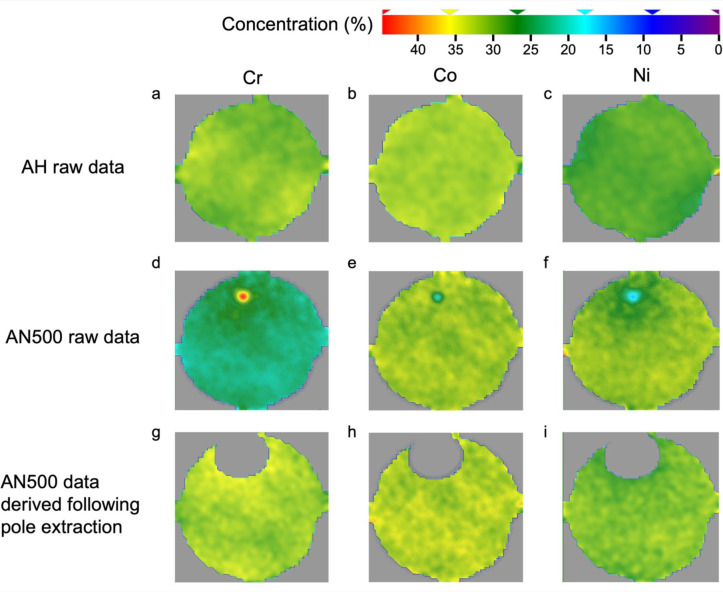
The concentration map of AH and AN500 heat treatment conditions. (**a**-**c**) concentration map of AH condition, (**d**–**f**) concentration map of AN500, and (**g**–**i**) pole extraction process on the detector map of AN500 is applied to reduce the effect of preferential evaporation.

We generated concentration maps for Cr, Co and Ni from the raw APT data using AP Suite 6.1 to check for the potential of segregation to crystallographic poles or other effects. We calculated these concentration maps for both AH (Extended Data Fig. 10a–c) and AN500 (Extended Data Fig. 10d–f) heat treatment conditions (Extended Data Fig. 10). We found evidence of the preferential segregation of Cr near the {111} pole for the AN500 sample (Extended Data Fig. 10d). To eliminate the potential for this segregation to bias our SRO measurements, we removed a cylinder of the data (10 mm radius in detector; Extended Data Fig. 10g–i) centred at the {111} pole in AP Suite 6.1 using a technique introduced elsewhere^54^. Our SRO algorithms were applied to the new, filtered data to remove any effects from segregation to poles.

### Glossary explanation

Spatial resolution, the spatial resolution of the APT instrument

Spatial noise, the simulation of APT spatial resolution using Gaussian noise

Modulation change, a modification in certain characteristics, such as amplitude, frequency or repetition pattern in the APT–SDM curves

True SRO, value of SRO returned from simulations when non-random distributions embedded in a perfect crystal (no detection loss and noise)

Measured SRO, value of SRO returned from simulations when analysing a degraded simulation (with detection noise and noise)

Reconstituted SRO, measured SRO times related correction factors

Experimentally measured SRO, value of SRO received from the APT experiments

## Online content

Any methods, additional references, Nature Portfolio reporting summaries, source data, extended data, supplementary information, acknowledgements, peer review information; details of author contributions and competing interests; and statements of data and code availability are available at 10.1038/s41563-024-01912-1.

### Supplementary information

Supplementary Information Supplementary Discussions 1–3, Tables 1–6 and Figs. 1–8.

### Source data

Source Data Fig. 2 Specific values of the imposed SRO parameters for Fig. 2b and the related maximum 95% confidence levels of the different SRO values.

Source Data Fig. 4 Data points correspond to the pairwise permutations for Fig. 4a and the models embedded with arbitrary true SRO values for Fig. 4b–d.

Source Data Fig. 5 Original experimental data for Fig. 5c.

## Data Availability

The core data used to evaluate the conclusions can be found in the Article, Extended Data Figs. 1–10 and Supplementary Information. Raw data are available from the corresponding author upon reasonable request. Source data are provided with this paper.

## Code availability

The steps and algorithms employed in this study are systematically outlined in the Methods. Custom models developed for this study are available from the corresponding author upon reasonable request.

## References

[1] Cohen, J. B. & Fine, M. E. Some aspects of short-range order. *J. Phys. Radium* **23**, 749–762 (1962). 10.1051/jphysrad:019620023010074901

[2] Schönfeld, B. Local atomic arrangements in binary alloys. *Prog. Mater. Sci.* **44**, 435–543 (1999). 10.1016/S0079-6425(99)00005-5

[3] Schön, C. G. On short-range order strengthening and its role in high-entropy alloys. *Scr. Mater.* **196**, 113754 (2021). 10.1016/j.scriptamat.2021.113754

[4] Lei, Z. et al. Enhanced strength and ductility in a high-entropy alloy via ordered oxygen complexes. *Nature* **563**, 546–550 (2018). 30429610 10.1038/s41586-018-0685-y

[5] George, E. P., Raabe, D. & Ritchie, R. O. High-entropy alloys. *Nat. Rev. Mater.* **4**, 515–534 (2019). 10.1038/s41578-019-0121-4

[6] Gludovatz, B. et al. A fracture-resistant high-entropy alloy for cryogenic applications. *Science* **345**, 1153–1158 (2014). 25190791 10.1126/science.1254581

[7] Gerold, V. & Karnthaler, H. P. On the origin of planar slip in f.c.c. alloys. *Acta Metall.* **37**, 2177–2183 (1989). 10.1016/0001-6160(89)90143-0

[8] Han, D., Guan, X. J., Yan, Y., Shi, F. & Li, X. W. Anomalous recovery of work hardening rate in Cu-Mn alloys with high stacking fault energies under uniaxial compression. *Mater. Sci. Eng. A* **743**, 745–754 (2019). 10.1016/j.msea.2018.11.103

[9] Niu, R. et al. Mechanical properties and deformation behaviours of submicron-sized Cu–Al single crystals. *Acta Mater.* **223**, 117460 (2022). 10.1016/j.actamat.2021.117460

[10] Wu, Y. et al. Short-range ordering and its effects on mechanical properties of high-entropy alloys. *J. Mater. Sci. Technol.* **62**, 214–220 (2021). 10.1016/j.jmst.2020.06.018

[11] Zhang, R. et al. Short-range order and its impact on the CrCoNi medium-entropy alloy. *Nature* **581**, 283–287 (2020). 32433617 10.1038/s41586-020-2275-z

[12] Chen, X. et al. Direct observation of chemical short-range order in a medium-entropy alloy. *Nature* **592**, 712–716 (2021). 33911276 10.1038/s41586-021-03428-z

[13] Guo, L. et al. Short-range ordering induced serrated flow in a carbon contained FeCoCrNiMn high entropy alloy. *Micron* **126**, 102739 (2019). 31472329 10.1016/j.micron.2019.102739

[14] Yin, B., Yoshida, S., Tsuji, N. & Curtin, W. A. Yield strength and misfit volumes of NiCoCr and implications for short-range-order. *Nat. Commun.* **11**, 2507 (2020). 32427824 10.1038/s41467-020-16083-1 PMC7237450

[15] Inoue, K., Yoshida, S. & Tsuji, N. Direct observation of local chemical ordering in a few nanometer range in CoCrNi medium-entropy alloy by atom probe tomography and its impact on mechanical properties. *Phys. Rev. Mater.* **5**, 085007 (2021). 10.1103/PhysRevMaterials.5.085007

[16] Owen, L. R., Playford, H. Y., Stone, H. J. & Tucker, M. G. A new approach to the analysis of short-range order in alloys using total scattering. *Acta Mater.* **115**, 155–166 (2016). 10.1016/j.actamat.2016.05.031

[17] Zhang, F. X. et al. Local structure and short-range order in a NiCoCr solid solution alloy. *Phys. Rev. Lett.* **118**, 205501 (2017). 28581808 10.1103/PhysRevLett.118.205501

[18] Yang, Y. et al. Determining the three-dimensional atomic structure of an amorphous solid. *Nature* **592**, 60–64 (2021). 33790443 10.1038/s41586-021-03354-0

[19] Nomoto, K. et al. Medium-range order dictates local hardness in bulk metallic glasses. *Mater. Today* **44**, 48–57 (2021). 10.1016/j.mattod.2020.10.032

[20] Moniri, S. et al. Three-dimensional atomic structure and local chemical order of medium- and high-entropy nanoalloys. *Nature* **624**, 564–569 (2023). 38123807 10.1038/s41586-023-06785-z

[21] Williams, D. B. & Carter, C. B. *Transmission Electron Microscopy: A Textbook for Materials Science* Vol. 2 (Springer Science & Business Media, 1996).

[22] Miller, M. K. & Forbes, R. G. *Atom-Probe Tomography: The Local Electrode Atom Probe* (Springer, 2014).

[23] Zhu, S., Shih, H.-C., Cui, X., Yu, C.-Y. & Ringer, S. P. Design of solute clustering during thermomechanical processing of AA6016 Al–Mg–Si alloy. *Acta Mater.* **203**, 116455 (2021). 10.1016/j.actamat.2020.10.074

[24] Newman, R. W., Sanwald, R. C. & Hren, J. J. A method for indexing field ion micrographs. *J. Sci. Instrum.* **44**, 828–830 (1967). 10.1088/0950-7671/44/10/302

[25] Suram, S. K. & Rajan, K. Calibration of reconstruction parameters in atom probe tomography using a single crystallographic orientation. *Ultramicroscopy* **132**, 136–142 (2013). 23507030 10.1016/j.ultramic.2013.02.013

[26] De Geuser, F. & Gault, B. Reflections on the projection of ions in atom probe tomography. *Microsc. Microanal.* **23**, 238–246 (2017). 28148309 10.1017/S1431927616012721

[27] Day, A. C., Ceguerra, A. V. & Ringer, S. P. Introducing a crystallography-mediated reconstruction (CMR) approach to atom probe tomography. *Microsc. Microanal.* **25**, 288–300 (2019). 30712521 10.1017/S1431927618015593

[28] Baraldi, A. N. & Enders, C. K. An introduction to modern missing data analyses. *J. Sch. Psychol.* **48**, 5–37 (2010). 20006986 10.1016/j.jsp.2009.10.001

[29] Gault, B., Moody, M. P., Cairney, J. M. & Ringer, S. P. *Atom Probe Microscopy* Vol. 160 (Springer, 2012).

[30] Vurpillot, F., Bostel, A. & Blavette, D. Trajectory overlaps and local magnification in three-dimensional atom probe. *Appl. Phys. Lett.* **76**, 3127–3129 (2000). 10.1063/1.126545

[31] Gault, B., Moody, M. P., Cairney, J. M. & Ringer, S. P. Atom probe crystallography. *Mater. Today* **15**, 378–386 (2012). 10.1016/S1369-7021(12)70164-5

[32] Cowley, J. M. An approximate theory of order in alloys. *Phys. Rev.* **77**, 669–675 (1950). 10.1103/PhysRev.77.669

[33] de Fontaine, D. The number of independent pair-correlation functions in multicomponent systems. *J. Appl. Crystallogr.* **4**, 15–19 (1971). 10.1107/S0021889871006174

[34] Ceguerra, A. V. et al. Short-range order in multicomponent materials. *Acta Cryst.* **A68**, 547–560 (2012). 10.1107/S0108767312025706 22893238

[35] Moody, M. P. et al. Atomically resolved tomography to directly inform simulations for structure–property relationships. *Nat. Commun.* **5**, 5501 (2014). 25407499 10.1038/ncomms6501

[36] Gault, B. et al. Origin of the spatial resolution in atom probe microscopy. *Appl. Phys. Lett.* **95**, 034103 (2009). 10.1063/1.3182351

[37] Gault, B. et al. Spatial resolution in atom probe tomography. *Microsc. Microanal.* **16**, 99–110 (2010). 20082732 10.1017/S1431927609991267

[38] Hyde, J. M., Marquis, E. A., Wilford, K. B. & Williams, T. J. A sensitivity analysis of the maximum separation method for the characterisation of solute clusters. *Ultramicroscopy* **111**, 440–447 (2011). 21227588 10.1016/j.ultramic.2010.12.015

[39] Jägle, E. A., Choi, P.-P. & Raabe, D. The maximum separation cluster analysis algorithm for atom-probe tomography: parameter determination and accuracy. *Microsc. Microanal.* **20**, 1662–1671 (2014). 25327827 10.1017/S1431927614013294

[40] Geiser, B. P., Kelly, T. F., Larson, D. J., Schneir, J. & Roberts, J. P. Spatial distribution maps for atom probe tomography. *Microsc. Microanal.* **13**, 437–447 (2007). 18001510 10.1017/S1431927607070948

[41] Gault, B. et al. Reflections on the spatial performance of atom probe tomography in the analysis of atomic neighborhoods. *Microsc. Microanal.* **28**, 1116–1126 (2022). 10.1017/S1431927621012952 34666868

[42] De Geuser, F. & Gault, B. Metrology of small particles and solute clusters by atom probe tomography. *Acta Mater.* **188**, 406–415 (2020). 10.1016/j.actamat.2020.02.023

[43] Champness, P. E. *Electron diffraction in the Transmission Electron Microscope* (Garland Science, 2020).

[44] Cui, X.-Y. & Ringer, S. P. On the nexus between atom probe microscopy and density functional theory simulations. *Mater. Charact.* **146**, 347–358 (2018). 10.1016/j.matchar.2018.05.015

[45] Li, Y. et al. Convolutional neural network-assisted recognition of nanoscale L1_2_ ordered structures in face-centred cubic alloys. *npj Comput. Mater.* **7**, 8 (2021). 10.1038/s41524-020-00472-7

[46] Li, Y. et al. Quantitative three-dimensional imaging of chemical short-range order via machine learning enhanced atom probe tomography. *Nat. Commun.* **14**, 7410 (2022). 10.1038/s41467-023-43314-y PMC10654683 37973821

[47] Ji, Z., Li, T. & Yaghi, O. M. Sequencing of metals in multivariate metal-organic frameworks. *Science* **369**, 674–680 (2020). 32764067 10.1126/science.aaz4304

[48] Muniandy, Y. et al. Compositional variations in equiatomic CrMnFeCoNi high-entropy alloys. *Mater. Charact.* **180**, 111437 (2021). 10.1016/j.matchar.2021.111437

[49] Chen, Y.-S. et al. Observation of hydrogen trapping at dislocations, grain boundaries, and precipitates. *Science* **367**, 171–175 (2020). 31919217 10.1126/science.aaz0122

[50] Breen, A. J., Theska, F., Lim, B., Primig, S. & Ringer, S. P. Advanced quantification of the site-occupancy in ordered multi-component intermetallics using atom probe tomography. *Intermetallics* **145**, 107538 (2022). 10.1016/j.intermet.2022.107538

[51] Warren, B. E. X‐ray determination of the structure of liquids and glass. *J. Appl. Phys.* **8**, 645–654 (1937). 10.1063/1.1710241

[52] Vurpillot, F., Bostel, A., Cadel, E. & Blavette, D. The spatial resolution of 3D atom probe in the investigation of single-phase materials. *Ultramicroscopy* **84**, 213–224 (2000). 10945331 10.1016/S0304-3991(00)00035-8

[53] Moody, M. P., Gault, B., Stephenson, L. T., Haley, D. & Ringer, S. P. Qualification of the tomographic reconstruction in atom probe by advanced spatial distribution map techniques. *Ultramicroscopy* **109**, 815–824 (2009). 19362421 10.1016/j.ultramic.2009.03.016

[54] Stephenson, L. T., Moody, M. P., Liddicoat, P. V. & Ringer, S. P. New techniques for the analysis of fine-scaled clustering phenomena within atom probe tomography (APT) data. *Microsc. Microanal.* **13**, 448–463 (2007). 18001511 10.1017/S1431927607070900

