# Supplementary material for: Quantifying short-range order using atom probe tomography
Source: Nat Mater. 2024 Jul 2;23(9):1200–7. doi: 10.1038/s41563-024-01912-1 (PMC11364508; doi:10.1038/s41563-024-01912-1)
Supplement: Supplementary file 1 — Supplementary Discussions 1–3, Tables 1–6 and Figs. 1–8. [file 41563_2024_1912_MOESM1_ESM.pdf]

nature materials

Supplementary information https://doi.org/10.1038/s41563-024-01912-1

Quantifying short-range order using atom probe tomography

In the format provided by the authors and unedited

Supplementary Discussion 1

Discussion of SRO in homogenized and annealed CoCrNi Alloys

The homogenised AH state was determined to be closer to a state of near random solid solution. By comparison, the heat treated AN500 sample exhibits clear evidence of SRO across certain combinations of elemental species. This allows comparisons to other approaches and to other computational simulations. For example, Tsai et al. [1] suggested that the binary mixing enthalpies for different pairs are such that Co and Ni are easily mixed in a random manner, while Cr tends to have a repulsive interaction with both Co and Ni. Our experimental SRO analyses are consistent with this proposition. Medium temperature long term ageing facilitates the formation of Cr-Cr clusters and Ni-Ni co-clusters. However, Cr-Ni pairs repel each other, and the Co-Cr pairs were distributed almost randomly. There is considerable discussion in the literature about how the materials processing route is expected to be the dominant factor influencing the degree of SRO [2, 3, 4, 5]. Zhang et al. [3] have reported SRO after annealing at the relatively high temperature of 1000 °C, followed by furnace cooling. Inoue et al. [4] reported SRO in CoCrNi after annealing at 700 °C for 384 h and suggested that this was a precursor phenomenon for L12 type ordering. Similar Cr-Cr pair changes to the elemental distributions were found in the more complex CoCrFeNiMn alloy [6]. There, annealing at 500 °C and 700 °C for 500 days resulted in the enrichment of Cr atoms and the eventual precipitation of a Cr-rich body-centred cubic phase at 500 °C and σ phase precipitation at 700 °C. This is broadly consistent with our finding of Cr-Cr clustering reaction, but we do not comment on the potential for Cr-rich L12 precipitation. Further, we expect that different annealing temperatures will drive different SRO behaviours [5].

Supplementary Discussion 2

Discussion of the current method and its limitations

Since the reconstitution procedure introduced here was based on estimating the detection loss and spatial resolution, some variance in the predicted SRO values is to be expected between instruments. Moreover, the relationship between SRO values and detector loss is alloy dependent. In equi-atomic ternary alloys, SRO diminishes with decreasing detector efficiency while it was previously shown to remain similar in a dilute binary system[7]. Additionally, the SRO parameters calculated from the experimental datasets were taken from the results for the first 7NN, which represents the atoms present in the first atomic shell distance from each reference atom in an FCC lattice. In the MEAs studied here, the 1st and 2nd shell distances were ~0.25 nm and ~0.36 nm, respectively. However, the spatial noise in the x-y direction was estimated to be $\sigma_{x,y-}$ = ~0.25 nm (also supported by Supplementary Fig. 8). The influence of inter-shell crossover due to spatial noise was simplified and the following 2nd and 3rd shells were assumed to be near random in the subsequent reconstitution process presented in Figure 5. Notwithstanding these assumptions, the approach appears to work. Understanding the relatively simple FCC ternary alloy system studied here can set out a pathway for the development of more general reconstitution procedures[8]. We suggest that the simulation procedure introduced here can be applied to generate correction factors and a reconstitution procedure for any alloy composition, enabling ranging of the go/no-go regimes for the quantification of SRO, depending on instrumental performance. The present work allows an instrument-specific assessment of the capacity for SRO measurements based on spatial resolution and detection efficiency characteristics of the particular atom probe microscope. This is akin to electron microscopy where different electron optical configurations enable certain imaging over other modes. Here for example, one might expect that atom probes containing a reflectron have merit for high mass resolution but the degraded spatial resolution expected with the additional lensing and the degraded detection efficiency from additional mesh inserts will be to the detriment of SRO measurements.

Supplementary Discussion 3

Discussion of current advances in SRO measurements

This method was used to demonstrate that heat treatments can indeed generate changes in the degree of SRO in this system. Given the discussion and debate on these topics in the M/HEAs literature, the notion of using APT to measure SRO is a significant advance. Two classes of issues were considered to achieve this measurement. The primary or first order issues relate to the instrument—specifically finite detection efficiency and ion trajectory uncertainties. The secondary issues relate to the accuracy of the tomographic reconstruction and could be addressed by acquiring high quality data and applying crystallography-informed calibrations. The primary issues were addressed using a data science approach involving multiple atomistic simulations of both random and embedded SRO values to devise corrections to the SRO values for the detection efficiency and trajectory uncertainty. The SRO values measured from experimental APT data could then be used to as inputs to initialise a reconstitution process to determine the true SRO embedded in the sample. Our simulation framework enables the determination of when SRO can and cannot be measured using an atom probe microscope of given instrumental performance, and for a given alloy composition. The simulations provided here enable detection efficiency, lateral and in-depth spatial noise and alloy composition to serve as inputs to determine the threshold values of SRO that are measurable in APT.

We suggest that the quantification of the SRO in multicomponent equiatomic systems such as the M/HEAs studied here offers a new way to characterise and ultimately control atomic-scale microstructure of these materials. For example, navigation of the thermomechanical processing of these alloys can be grounded in SRO assessments.

Supplementary Table 1 The specific values of the imposed SRO parameters for Figure 2b and the related maximum 95% confidence levels (CL) of different SRO values.

**SRO (α)**	~0.1	~0.075	~0.050	~0.025	~0.01	~0.001
**95% CL**	0.00026	0.00019	0.00029	0.00026	0.00023	0.00018
**SRO (α)**	~-0.1	~-0.075	~-0.050	~-0.025	~-0.01	~-0.001
**95% CL**	0.00026	0.00026	0.00028	0.00021	0.00028	0.00022

Supplementary Table 2 The data points correspond to the pairwise permutations for Figure 4a.

**Point**	**1**	**2**	**3**	**4**	**5**	**6**	**7**	**8**	**9**	**10**
**SRO (α)**	0.1	0.075	0.05	0.025	0.015	0.005	0.003	0.001	0.00075	0.00012

Supplementary Table 3 The models embedded with arbitrary true SRO values for Figure 4b-d.

**Pair**	**CoCo**	**CoCr**	**CoNi**	**CrCo**	**CrCr**	**CrNi**	**NiCo**	**NiCr**	**NiNi**
**High α (Fig. 4b)**	-0.015	0.095	-0.08	0.095	-0.075	-0.02	-0.08	-0.02	0.103
**Medium α (Fig. 4c)**	0.002	0.002	-0.004	0.002	0.003	-0.006	-0.004	-0.006	0.010
**Low α (Fig. 4d)**	0.00001	-0.0001	0.0001	-0.0001	0.0012	-0.001	0.0001	-0.001	0.001

Supplementary Table 4 The original experimental data for Figure 5c.

	AH		AN500	
	Average	Standard deviation	Average	Standard deviation
CoCo	-0.00069	0.00020	0.00138	0.00067
CoCr	0.00047	0.00036	-0.00077	0.00011
CoNi	0.00021	0.00054	-0.00043	0.00066
CrCo	0.00065	0.00093	-0.00090	0.00032
CrCr	0.00071	0.00047	0.00385	0.00017
CrNi	-0.00131	0.00136	-0.00359	0.00054
NiCo	-0.00027	0.00034	-0.00029	0.00048
NiCr	-0.00231	0.00091	-0.00345	0.00005
NiNi	0.00250	0.00062	0.00424	0.00100

Supplementary Table 5 ICP-AES results for the CoCrNi ingot derived from weight percentages.

at. %	Cr	Co	Ni	Others
Ingot	32.51	33.75	33.32	0.26

Supplementary Table 6 The relationship between different SRO parameters.

	WC-SRO	PM-SRO	GM-SRO	Current SRO
**Formulae**	$\alpha_{AB}^{m} = 1 - \frac{P_{AB}}{X_B}$	$\alpha_{AB}^{m} = \frac{P_{AB} - X_B}{\delta - X_B}$	$\alpha_{AB}^{m} = -1^{(1+\delta)} \times \frac{P_{AB} - X_B}{\delta - X_B}$	$\alpha_{AB}^{m} = \frac{P_{AB} - X_B}{X_B}$
**if A = B, $\boldsymbol{\delta = 1}$, if A ≠ B, $\boldsymbol{\delta = 0}$.**				

Supplementary Fig. 1 The standard deviation plots of Figure 2c-2f.

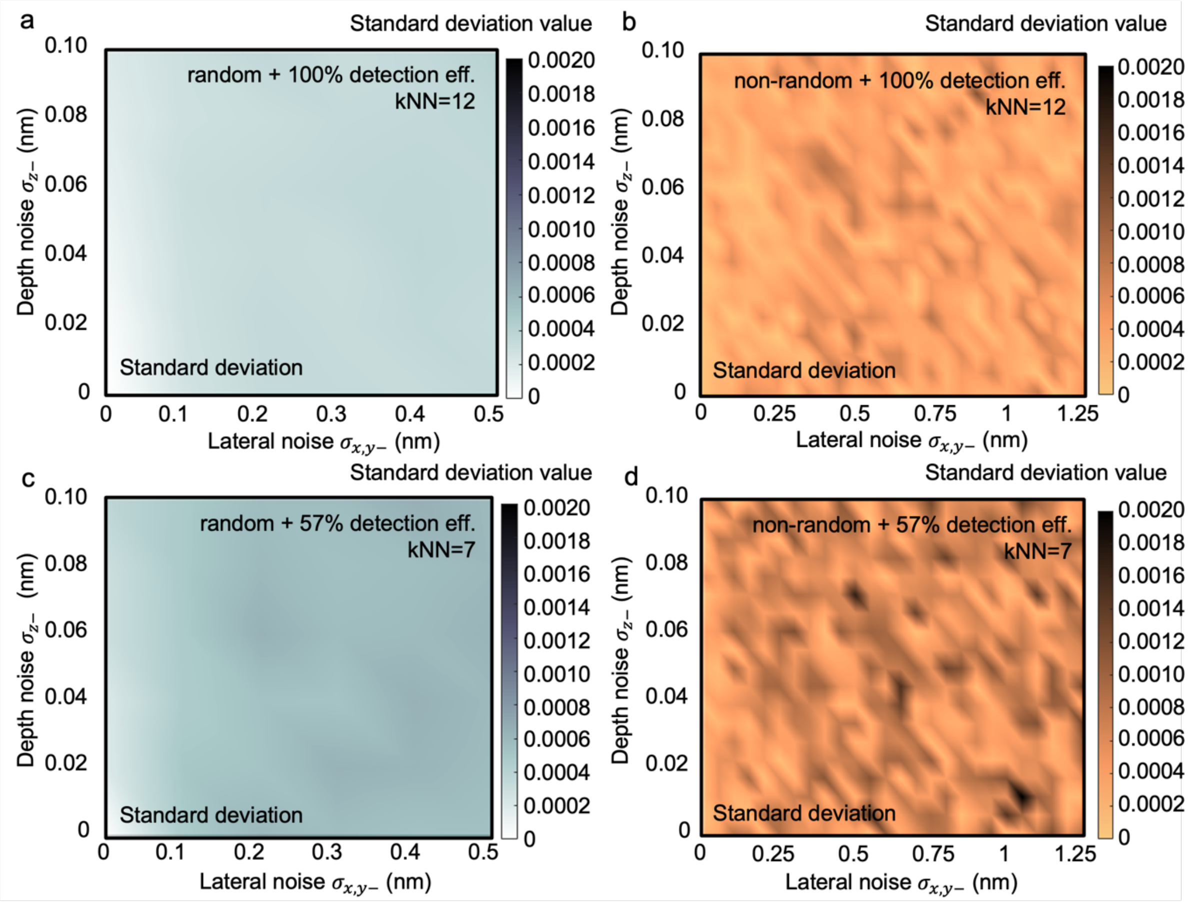

Supplementary Fig. 2 A reconstitution process to determine true SRO with different resolutions. (a) Measured SRO values (red) versus the true SRO values embedded in the simulated model of a CoCrNi MEA. This enables the determination of a correction factor, β, that accounts for the combined effects of the detection loss (57% detection rate) and the limited spatial resolution ($\sigma_{x,y-}$ = 0.50 nm, σ_{z-} = 0.1 nm) for each value of SRO for this alloy system (green). The SRO values for random simulations ranged $|\alpha| \leq 0.00022$. This correction factor separated into two regimes: a sharp tendency for this quotient to tend β → ~35 when there was little or no SRO, and a flat region where there exists a medium level of SRO, such that for α > 0.0016, ~7.1 ≤ β ≤ ~19.1. Comparison of the high (b), medium (c) and low (d) input true SRO values (black) to the reconstituted SRO values (blue). The 95% confidence intervals are provided. 57% of the data is simulated 100 times using the random labelling method with around 4 million atoms and SRO is measured for kNN = 7 to range the random values (violet). Data are presented as the average of the reconstituted SRO value for each pair +/- their 95% confidence region. The fidelity of the reconstitution process was preserved for the high and medium input SRO values (Supplementary Figs. 2b-c), but not for the low input values (Supplementary Fig. 2d).

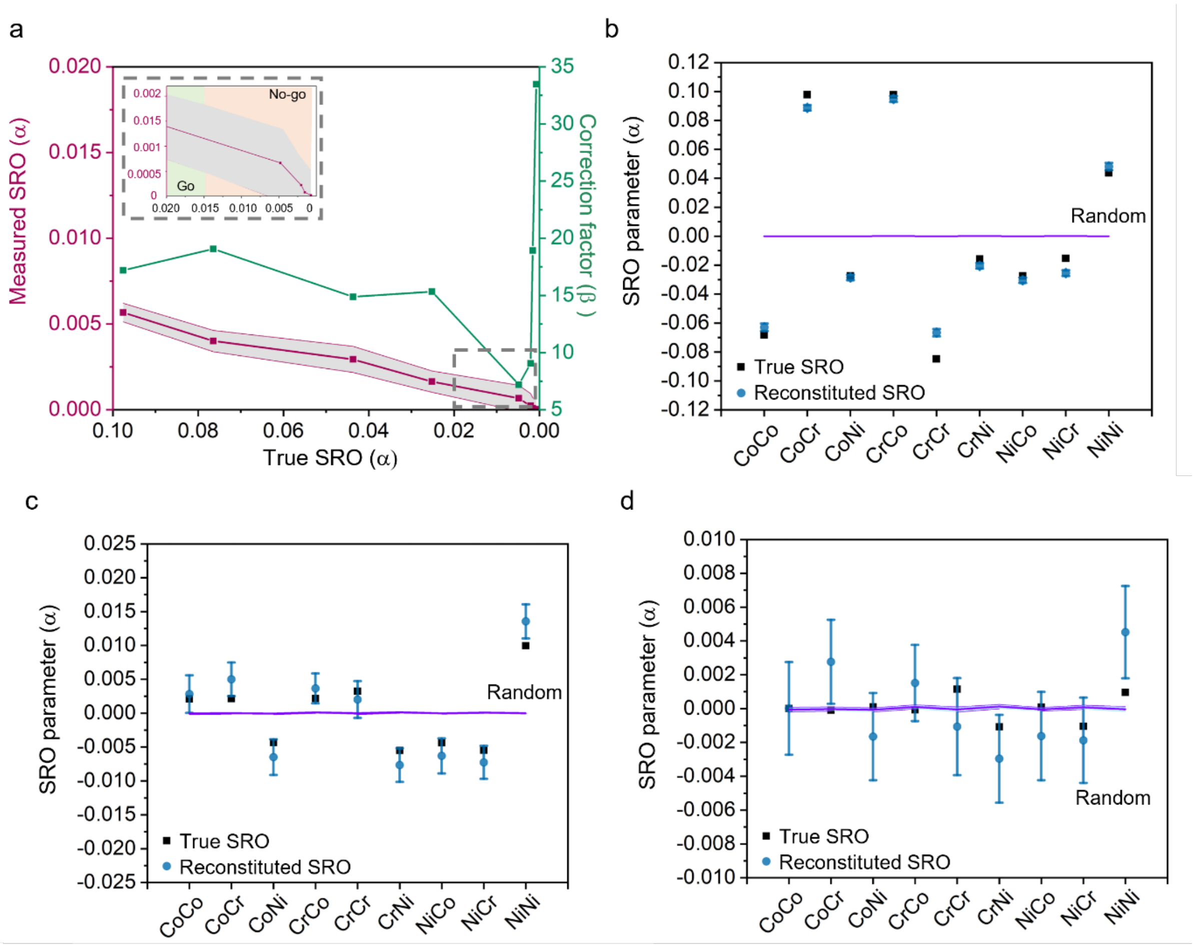
a
Measured SRO (α)
0.020
0.015
0.010
0.005
0.000
True SRO (α)
0.10
0.08
0.06
0.04
0.02
0.00
Correction factor (β)
35
30
25
20
15
10
5
No-go
Go
0.002
0.015
0.001
0.0005
0
0.020
0.015
0.010
0.005
0
b
SRO parameter (α)
0.12
0.10
0.08
0.06
0.04
0.02
0.00
-0.02
-0.04
-0.06
-0.08
-0.10
-0.12
Random
True SRO
Reconstituted SRO
CoCo
CoCr
CoNi
CrCo
CrCr
CrNi
NiCo
NiCr
NiNi
c
SRO parameter (α)
0.025
0.020
0.015
0.010
0.005
0.000
-0.005
-0.010
-0.015
-0.020
-0.025
Random
True SRO
Reconstituted SRO
CoCo
CoCr
CoNi
CrCo
CrCr
CrNi
NiCo
NiCr
NiNi
d
SRO parameter (α)
0.010
0.008
0.006
0.004
0.002
0.000
-0.002
-0.004
-0.006
-0.008
-0.010
Random
True SRO
Reconstituted SRO
CoCo
CoCr
CoNi
CrCo
CrCr
CrNi
NiCo
NiCr
NiNi

Supplementary Fig. 3 A reconstitution process to determine true SRO with different resolutions. (a) Measured SRO values (red) versus the true SRO values embedded in the simulated model of a CoCrNi MEA. This enables the determination of a correction factor, β, that accounts for the combined effects of the detection loss (57% detection rate) and the limited spatial resolution ($\sigma_{x,y-}$ = 1 nm, σ_{z-} = 0.1 nm) for each value of SRO for this alloy system (green). Comparison of the high (Supplementary Fig. 3b) input true SRO values (black) to the reconstituted SRO values (blue). The 95% confidence intervals are provided. 57% of the data is simulated 100 times using the random labelling method with around 4 million atoms and SRO is measured for kNN = 7 to range the random values (violet). Data are presented as the average of the reconstituted SRO value for each pair +/- their 95% confidence region. The trend of SRO after the reconstitution process was preserved for the high input SRO values (Supplementary Fig. 3b), but not for the medium or low input values.

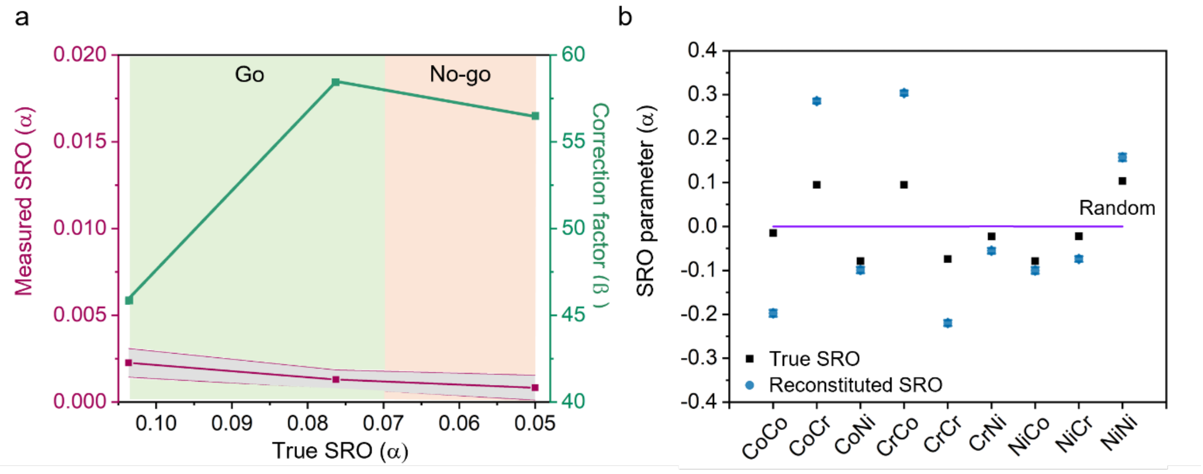

Supplementary Fig. 4 The XRD (Mo source) results of both conditioned samples.

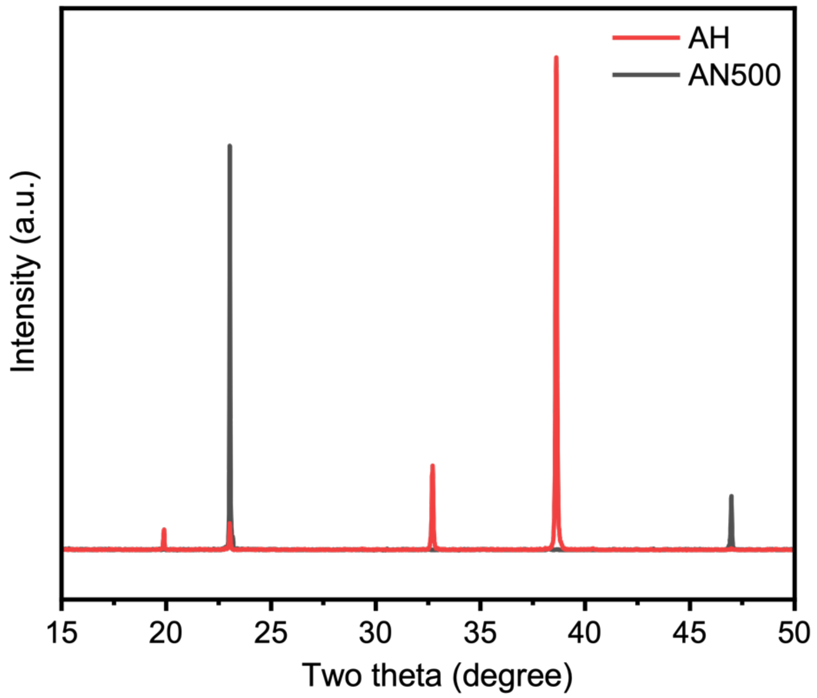

Supplementary Fig. 5 The EDXS results of AN500 sample showing no atomic preference.

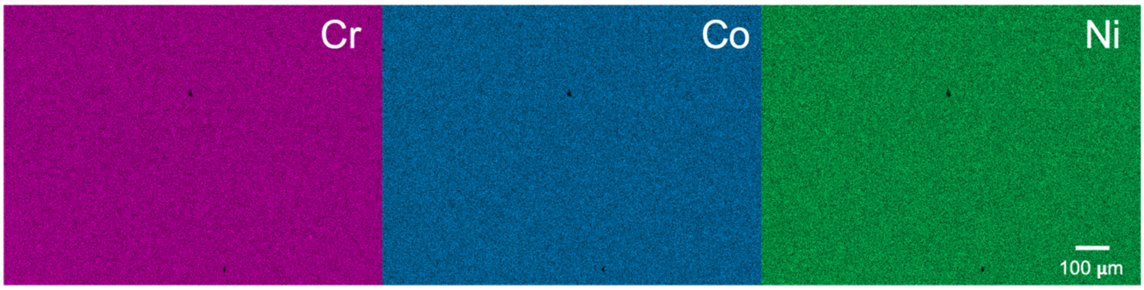

Supplementary Fig. 6 Resolution analysis using a standard pure aluminum sample. (a) Three-dimensional APT reconstruction of standard pure aluminum. (b) A 2D spatial distribution map (SDM) illustrating APT's lateral resolution capability to detect atomic distances smaller than 0.28 nm. (c) Demonstration of the z-resolution capability in our APT experiment, evidenced by the sharpness of the peak distribution in the 1D-SDM.

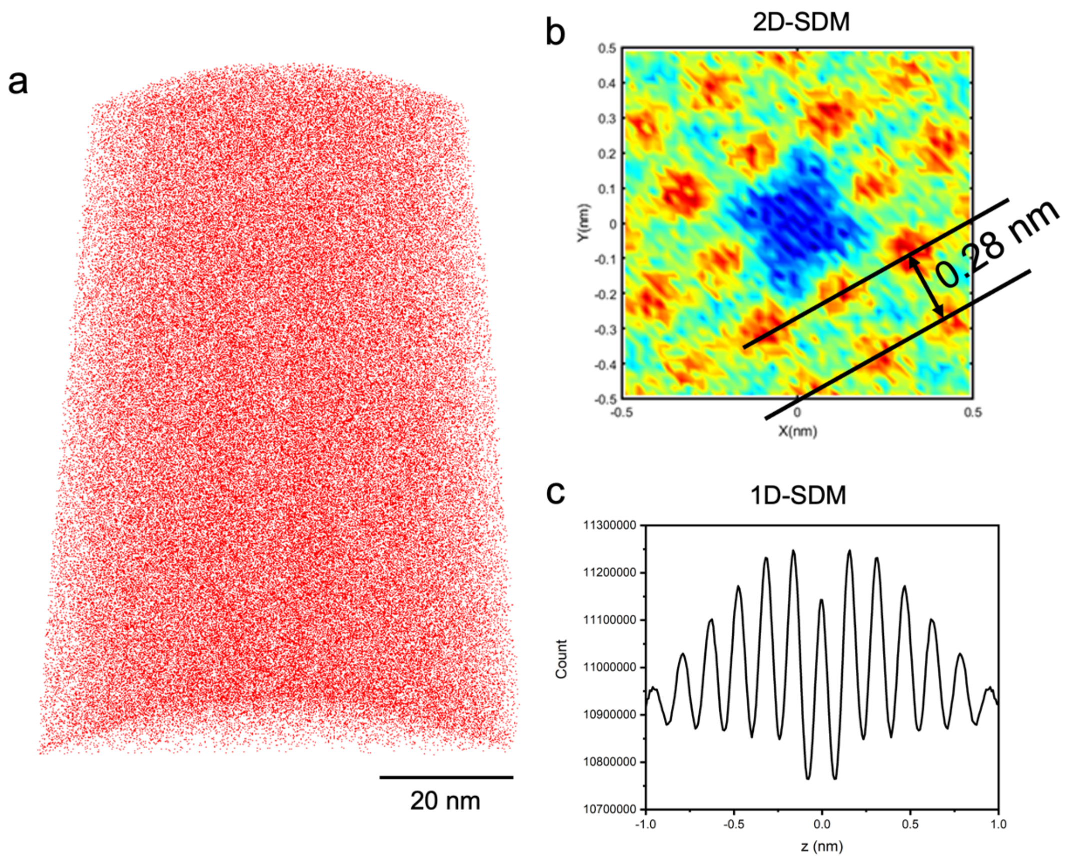

Supplementary Fig. 7 Comparison between random labelling and experimental results. (a) illustrates one instance of random labelling versus the experimental data. (b) compares ten random cases with the experimental data. The shaded areas highlight the regions of difference between the experimental results and the random cases, specifically within the first 40 nearest neighbours (40NN) atoms.

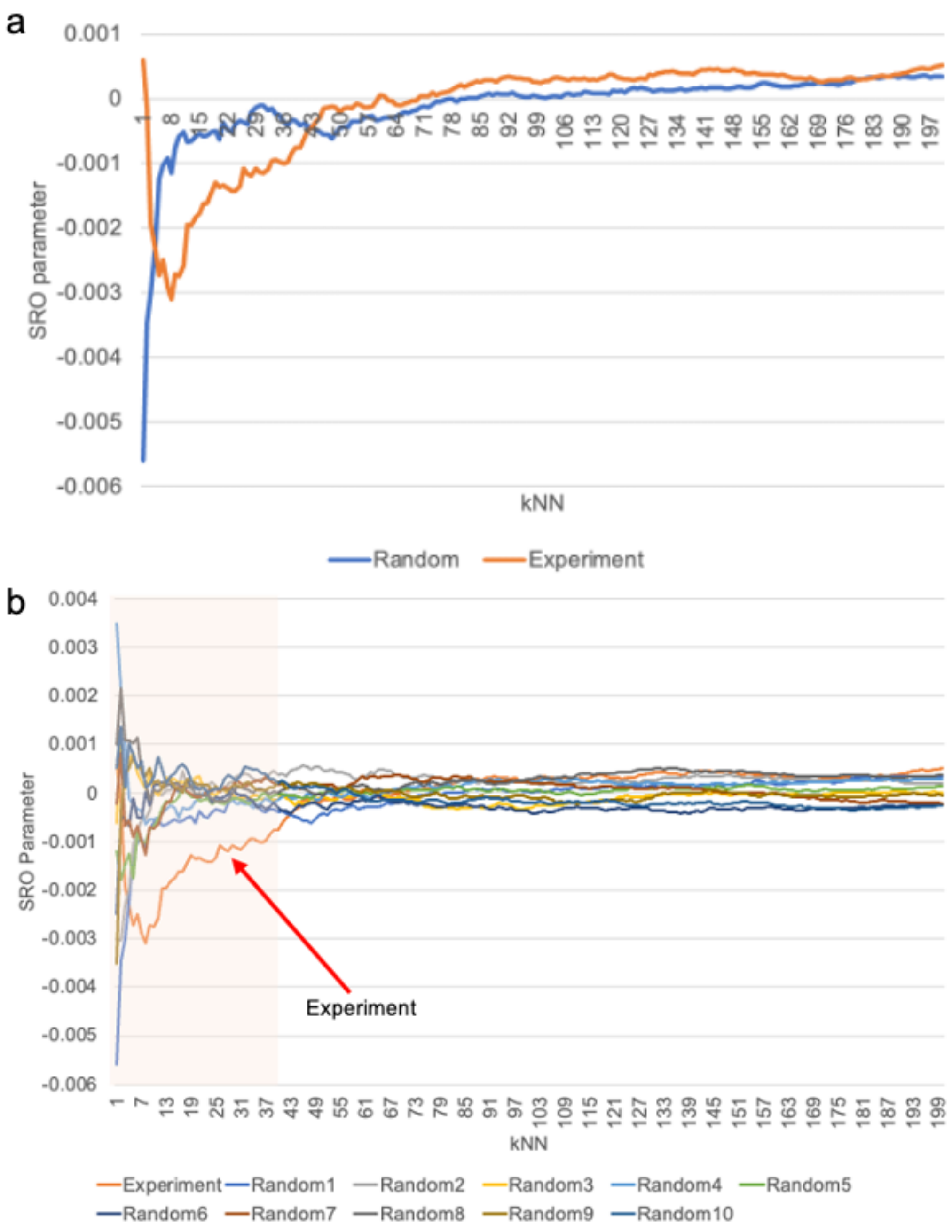

Supplementary Fig. 8 D-1NN distributions between atoms initially nearest neighbours. Atoms located at the same depth in the input data of (a) simulated data, (b) experiment data.

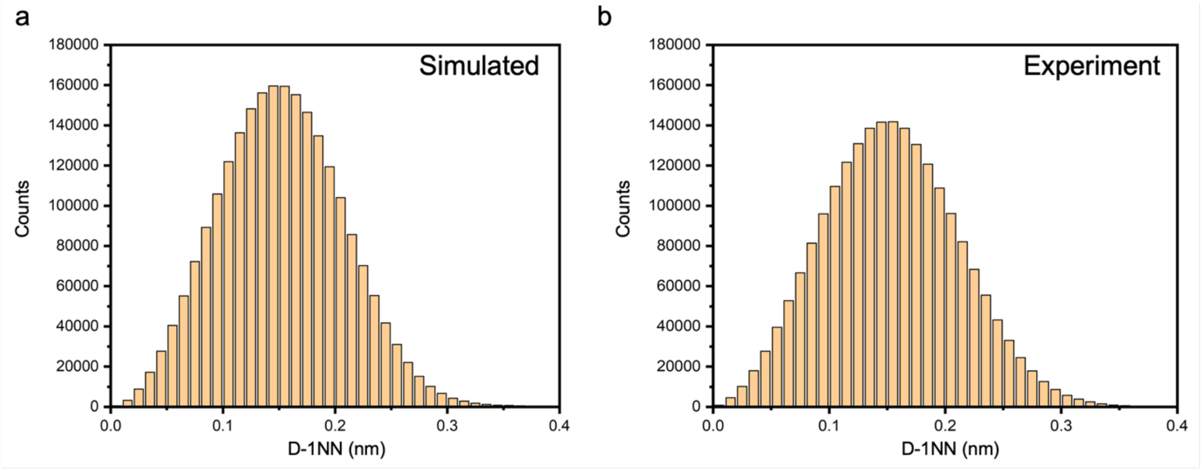

In Supplementary Fig. 8, we plot the D-1NN distances of our simulated data, with a standard deviation of 0.25 nm for noise added in the x,y-plane and 0.1 nm in the z-direction, alongside the experimental data from the {110} AH sample. We found that the simulated data yields a FWHM (Full Width at Half Maximum) of D-1NN distances of 0.139 nm (Supplementary Fig. 8a), compared to 0.145 nm for the experimental data (Supplementary Fig. 8b). This indicates that our D-1NN condition is comparable/better to that reported in the Gault, Klaes et al. paper [9]. Importantly, it also demonstrates that meaningful SRO values can be measured at these resolution levels.

References

1. Tsai KY, Tsai MH, Yeh JW. Sluggish diffusion in Co–Cr–Fe–Mn–Ni high-entropy alloys. *Acta Materialia* 2013, **61**(13)**:** 4887-4897.

2. Hsiao H-W, Feng R, Ni H, An K, Poplawsky JD, Liaw PK*, et al.* Data-driven electron-diffraction approach reveals local short-range ordering in CrCoNi with ordering effects. *Nature Communications* 2022, **13**(1)**:** 6651.

3. Zhang R, Zhao S, Ding J, Chong Y, Jia T, Ophus C*, et al.* Short-range order and its impact on the CrCoNi medium-entropy alloy. *Nature* 2020, **581**(7808)**:** 283-287.

4. Inoue K, Yoshida S, Tsuji N. Direct observation of local chemical ordering in a few nanometer range in CoCrNi medium-entropy alloy by atom probe tomography and its impact on mechanical properties. *Physical Review Materials* 2021, **5**(8)**:** 085007.

5. Li L, Chen Z, Kuroiwa S, Ito M, Yuge K, Kishida K*, et al.* Evolution of short-range order and its effects on the plastic deformation behavior of single crystals of the equiatomic Cr-Co-Ni medium-entropy alloy. *Acta Materialia* 2023, **243:** 118537.

6. Otto F, Dlouhý A, Pradeep KG, Kuběnová M, Raabe D, Eggeler G*, et al.* Decomposition of the single-phase high-entropy alloy CrMnFeCoNi after prolonged anneals at intermediate temperatures. *Acta Materialia* 2016, **112:** 40-52.

7. de Fontaine D. The number of independent pair-correlation functions in multicomponent systems. *Journal of Applied Crystallography* 1971, **4**(1)**:** 15-19.

8. Kelly TF, Gorman BP, Ringer SP. *Atomic-Scale Analytical Tomography: Concepts and Implications*. Cambridge University Press: Cambridge, 2022.

9. Gault B, Klaes B, Morgado FF, Freysoldt C, Li Y, De Geuser F*, et al.* Reflections on the spatial performance of atom probe tomography in the analysis of atomic neighborhoods. *Microscopy and Microanalysis* 2022, **28**(4)**:** 1116-1126.
